# Oldest Known Pantherine Skull and Evolution of the Tiger

**DOI:** 10.1371/journal.pone.0025483

**Published:** 2011-10-10

**Authors:** Ji H. Mazák, Per Christiansen, Andrew C. Kitchener

**Affiliations:** 1 Shanghai Science and Technology Museum, Shanghai, People's Republic of China; 2 Department of Biotechnology, Chemistry, and Environmental Engineering, University of Aalborg, Aalborg, Denmark; 3 National Museums Scotland, Edinburgh, United Kingdom; University College London, United Kingdom

## Abstract

The tiger is one of the most iconic extant animals, and its origin and evolution have been intensely debated. Fossils attributable to extant pantherine species-lineages are less than 2 MYA and the earliest tiger fossils are from the Calabrian, Lower Pleistocene. Molecular studies predict a much younger age for the divergence of modern tiger subspecies at <100 KYA, although their cranial morphology is readily distinguishable, indicating that early Pleistocene tigers would likely have differed markedly anatomically from extant tigers. Such inferences are hampered by the fact that well-known fossil tiger material is middle to late Pleistocene in age. Here we describe a new species of pantherine cat from Longdan, Gansu Province, China, *Panthera zdanskyi* sp. nov. With an estimated age of 2.55–2.16 MYA it represents the oldest complete skull of a pantherine cat hitherto found. Although smaller, it appears morphologically to be surprisingly similar to modern tigers considering its age. Morphological, morphometric, and cladistic analyses are congruent in confirming its very close affinity to the tiger, and it may be regarded as the most primitive species of the tiger lineage, demonstrating the first unequivocal presence of a modern pantherine species-lineage in the basal stage of the Pleistocene (Gelasian; traditionally considered to be Late Pliocene). This find supports a north-central Chinese origin of the tiger lineage, and demonstrates that various parts of the cranium, mandible, and dentition evolved at different rates. An increase in size and a reduction in the relative size of parts of the dentition appear to have been prominent features of tiger evolution, whereas the distinctive cranial morphology of modern tigers was established very early in their evolutionary history. The evolutionary trend of increasing size in the tiger lineage is likely coupled to the evolution of its primary prey species.

## Introduction

The extant pantherine cats comprise a well supported clade of seven extant species and several fossil species primarily known from the Middle and Late Pleistocene [1], [2], and fossils attributable to all extant species-lineages are also Pleistocene (Calabrian-Tarantian) [2]–[4]. The earliest known *Panthera* fossils are from the transition between Early and Late Pliocene of East Africa with an estimated age of <3.8 Ma, corresponding to the latest Zanclean or early Piacenzian [2], [5]; these comprise maxillary and mandibular fragments, a few isolated tooth and postcranial elements of a lion-sized species and a leopard-sized species, but their taxonomic status is still open to question, although they have tentatively been attributed to *Panthera* cf. *leo* and *P*. cf. *pardus*, respectively [5]–[8]. It is possible that they are members of the stem-lineage leading to the leopard/lion crown clade [2], [8], which is well supported in phylogenetic analysis [1], [9].

The oldest member of the tiger lineage is traditionally considered to be *P. palaeosinensis* [10] and primitive tigers are inferred to have been morphologically similar to *P. palaeosinensis* [4], [11]. However, modern cladistic and morphometric studies do not support a close affinity to the tiger, and instead indicate a more basal position within the Pantherinae [1], [12]. Dating of *P. palaeosinensis* is uncertain but it is traditionally held to be Early Pleistocene or around the traditional Plio-Pleistocene boundary [3], [4], [11], [13]. The oldest known fossils definitively attributable to tigers comprise maxillary and mandibular fragments from the Lower Pleistocene (Calabrian) of Lantian, China, whereas the few largely complete skulls are all from the late Middle or Late Pleistocene [4], [14]–[20]. The geographical origin of the tiger has been much debated; it is believed to have originated either in north-central China [16], [17], southern China [21], or northern Siberia [20], [22]. Extant putative subspecies show morphological [4], [16], [17], [23], [24] and genetic [25] differences, and are inferred to have diverged much later at <100 KYA [25], [26].

**Figure 1:**
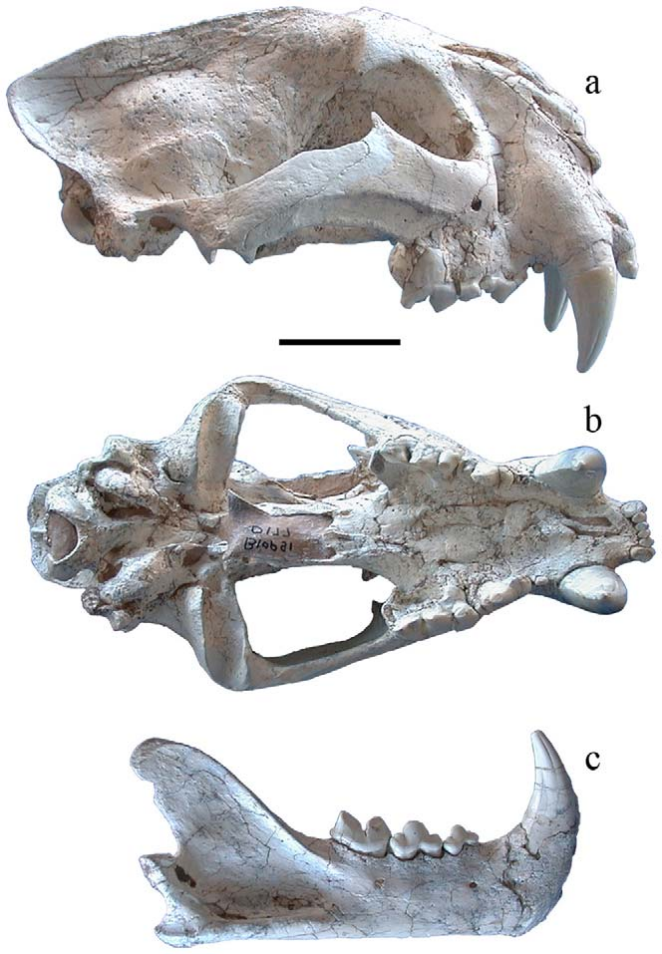
Holotype of *Panthera zdanskyi* sp. nov. BIOPSI 00177 (Babiarz Institute of Paleontological Studies) from the earliest Pleistocene of Longdan, Dongxiang County, Gansu Province, China in A, lateral; and B, ventral views; C, lateral view of mandible. The skull measures as follows (in mm): greatest skull length, 264.0; condylobasal length, 236.3; nasal length, 81.6; mandible length, 167.8; and mandible posterior height, 85.4. Dental measurements are as follows (in mm): C^1^ height, 56.0; C^1^ alveolar length, 24.0; C^1^ alveolar width, 16.7; P^4^ length, 31.7; P^4^ width, 17.0; P^3^ length, 22.0; P^3^ width, 10.0; P^2^ length, 5.5; C_1_ height, 40.5; C_1_; alveolar length, 21.9; C_1_ alveolar width, 13.0; M_1_ length, 24.6; M_1_ width, 10.7; P_4_ length, 21.7; P_4_ width; P_3_ length, 15.4; P_3_ width, 9.7.

A recently discovered and varied mammal fauna from the Lower Pleistocene in Longdan, Dongxiang County, Gansu Province of north-western China was announced in 2004 [27], and palaeomagnetic data have allowed an accurate dating at 2.55–2.16 MYA [27]. This is traditionally equivalent to the last stage (Gelasian, 2.588-1.806 MYA) of the Pliocene [28], [29]; however, recently, the Gelasian was re-assigned to the basal Pleistocene by the International Commission on Stratigraphy [30]. Among the fossils was a pantherine rostrum, which was assigned to *P palaeosinensis* based largely on size [27]. The recent discovery of a complete and well-preserved skull at Longdan demonstrates that the rostrum cannot be referred to *P. palaeosinensis*, but is a new species of jaguar-sized pantherine, which is morphologically far more tiger-like than *P. palaeosinensis* (Fig. 1). Predating known tiger fossils by at least half a million years, this discovery opens a new window on the origin and evolution of the tiger lineage, and also has significant implications for pantherine evolution in general.

## Results

### SYSTEMATIC PALAEONTOLOGY

Class MAMMALIA Linnaeus, 1758

Order CARNIVORA Bowdich, 1821

Family FELIDAE Fischer, 1817

Subfamily PANTHERINAE Pocock, 1917

Genus PANTHERA, Oken, 1816

*Panthera zdanskyi* sp. Nov (urn:lsid:zoobank.org:act:A7A75025-5E17-4CAA-B6FE-400301F8A57D)

#### Etymology

In recognition of the late Austrian paleontologist Dr. Otto A. Zdansky (1894–1988), who contributed greatly to our knowledge of Neogene Chinese fossil carnivorans.

#### Holotype

An almost complete skull and mandible (Babiarz Institute of Paleontological Studies B.I.O.P.S.I 00177). The Babiarz Institute of Paleontological Studies, Inc., in Mesa, Arizona, is a privately owned institute specializing in fossil cats, which has a number of other felid type specimens registered, e.g., the unusual saber-toothed felid *Xenosmilus hodsonae* [31].

#### Paratype

A rostrum, premaxilla and maxilla and much of the dentition (Institute of Vertebrate Paleontology and Paleoanthropology, Chinese academy of Science IVPP 13538), originally referred to *P. palaeosinensis* [27].

#### Type Locality

East slope of Longdan, south of Dongxiang Autonomous county, Gansu province, N.W. China.

#### Geological age and fauna

Specimens of *P. zdanskyi* were found in the Lower Pleistocene *Equus* fauna, which has been dated to 2.55–2.16 MA (Gelasian, basal-most Pleistocene).

### Diagnosis

A jaguar-sized pantherine with a robust skull; well developed cranial muscular crests; large, robust canines; long nasals relative to skull size, which extend posterior to the maxilla-frontal suture; heart-shaped narial aperture; robust mandible with straight ventral profile; proportionally large carnassials and large teeth overall; upper carnassial (P^4^) with a distinct ectoparastyle and well-developed protocone; lower carnassial (M_1_) with a well-defined talonid, and short and low paraconid and protoconid cusps relative to crown length; M_1_ with a large paraconid relative to the protoconid; P_4_ with large protoconid, nearly half of crown length.

### Description of the material

#### Holotype

The holotype consists of a well-preserved cranium and mandible (Fig. 1). The cranium is moderately latero-medially compressed and its left side is slightly dorso-ventrally flattened in the frontal-orbitial region and is slightly pushed upwards (about 10–15 mm) relative to the right side. The nasals are somewhat laterally compressed and slightly more beveled than would originally have been the case. The mandible has also suffered some lateral compression of the rami, but each ramus is in perfect condition, and the entire dentition is excellently preserved. The above implies that overall width measurements and further morphometric comparisons of three-dimensional aspects of the cranium are unreliable. However, morphometric comparisons of the lateral views of the specimen are feasible, especially the right side of the cranium and left mandibular ramus. Metric variables of the dentition and along the long axis of the cranium are reliable as these are not influenced by compaction.

Overall cranial morphology is typical of *Panthera* spp. The cranium is heavy and robust; the frontal-interorbital region is not noticeably vaulted; the sagittal crest is well developed such that the area behind the frontal elevation is less steeply sloped and the dorsal profile is fairly straight; the lambdoidal crests are well developed; and the neurocranial axis is nearly horizontal to the splanchnocranial axis. The facial part of the cranium is massive. Although compressed laterally, the nasals are evidently elongated and generally triangular in shape in dorsal perspective, narrowing posteriorly, and they clearly project posteriorly to the frontal processes of the maxillae; the nasal processes of the nasals (*processus nasalis ossis nasilis*) are long. The frontal-maxillary suture is acute and square-shaped. The infraorbital foramen is relatively larger than that of the paratype. There is a deep longitudinal depression in the frontal region. The zygomatic arches are massive, bearing strong lateral antero-posterior ridges for the *M. masseter profunda*. In ventral view, the posterior margin of the palate is V-shaped, and the longitudinal depressions on the palate are deeply marked.

The mandible is also robust and typically *Panthera*-like. The horizontal ramus is particularly massive with a nearly straight ventral profile, and the anterior symphysis is robust and moderately anteriorly inclined with a rounded anterior edge. The coronoid process is well developed and inclined posteriorly, the masseteric fossa reaches the anterior edge of M_1_ and is strongly excavated with a well developed crest along its antero-ventral margin, indicating powerful mandibular adductors.

The teeth are proportionally large, and the canines are conspicuously tall and robust, in particular compared with those of similar-sized leopards (*P. pardus*) and jaguars (*P. onca*). The upper carnassial (P^4^) has a distinct ectoparastyle and a well-developed protocone. The lower carnassial (M_1_) has a distinct talonid, and the paraconid and protoconid cusps are rather short and low compared to crown length; additionally, the paraconid is large relative to the protoconid.

#### Paratype

**Figure 2:**
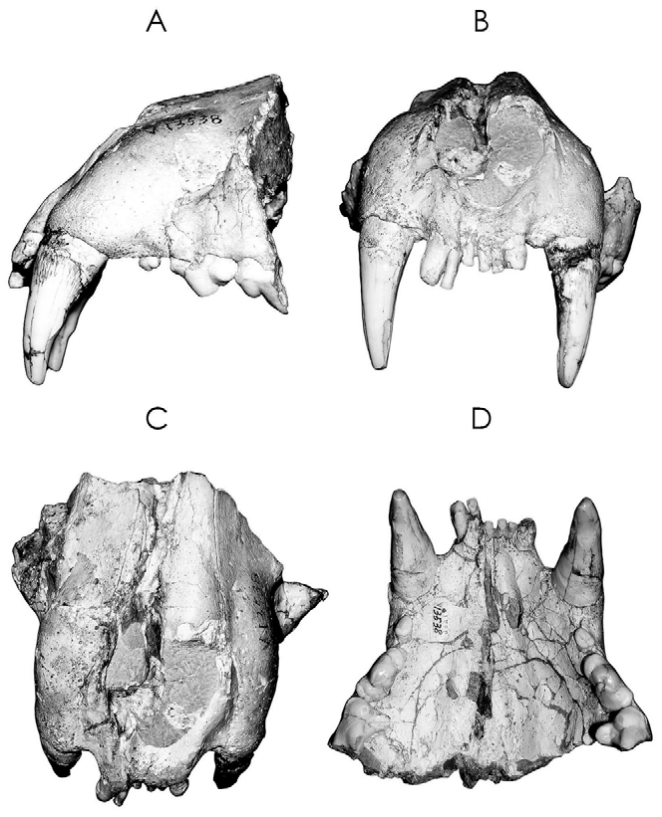
Paratype of *Panthera zdanskyi* sp. nov. IVPP13538.

A maxillary with I^1^-I^3^, C^1^, P^2^, P^3^ and the anterior part of P^4^ (Fig. 2). Originally referred to *P. palaeosinensis* [27], it is distinctly different from the type specimen of *P. palaeosinensis* but is nearly identical to the holotype of *P. zdanskyi* (Supporting Information Fig. S1). The maxillary is slightly dorso-ventrally flattened and its right side is pushed slightly anteriorly. It is intermediate in size between the maxillaries of large male leopards (*P. pardus*) and Sunda Island tigers (*P. t .sumatrae*, *P. t. sondaica*). The snout is relatively vertical and is massive at the root of the canines. The anterior narial aperture is heart-shaped with a narrow and tapered ventral area similar to that of *P. zdanskyi* and tigers. The infraorbital foramen appears to be relatively large and its shape closely matches those of *P. zdanskyi* and tigers. The palate is broad and short. Both I^1^ and I^2^ are small and I^3^ is distinctly (>50%) larger than I^1^ and I^2^. The canine is conspicuously large and robust, very similar to those of *P. zdanskyi* and tigers. Along its lateral aspect are two distinct longitudinal grooves. P^2^ is small, and P^3^ has a low anterior cusp (parastyle), a large main cusp (paracone), a low metastyle cusp, and a raised, thickened posterior cingulum. Only the anterior parts of P^4^ are preserved, but as far as can be observed, crown morphology is close to that of *P. zdanskyi* and tigers, such that there is a small, but distinct, ectoparastyle and a strongly developed protocone.

### Comparison with other *Panthera*

With a condylobasal skull length (CBL) of 236 mm, the type specimen of *P. zdanskyi* is similar in size to the smallest females of extant tiger subspecies (Supporting Information Fig. S2), but its overall morphology indicates that it was a male [32]. Typical of tigers [33], [34], the upper canine is well developed and robust, and its crown height is 23.7% of CBL. Compared to other *Panthera*, this is even higher than in extant tigers (0.166–0.230); and much higher than in jaguars (0.160–0.206), leopards (0.132–0.202), lions (0.141–0.185), *P. palaeosinensis* (0.200), and the Late Pleistocene *P. atrox* (0.150–0.186) and *P. spelaea* (0.168–0.186). This massive canine is also present in the paratype. Another characteristic trait of tigers is long nasals relative to skull size [12], [33], [34] and in *P. zdanskyi* the nasals are 34.5% of CBL; this is within the lower range for extant tigers (0.333–0.417); and at the extreme upper ranges in jaguars (0.275–0.346), leopards (0.296–0.347), and lions (0.287–0.357); and higher than in *P. atrox* (0.258–0.291) or *P. spelaea* (0.301–0.312). As noted above, the nasals project well posterior to the maxilla-frontal suture, another characteristic tiger trait [12], [33], [34], which is absent in *P. palaeosinensis*, where they are approximately at level with each other. The zygomatic arches are massive, and zygomatic height at the postorbital process is 14.3% of CBL; this is at the upper range of tigers (0.095–0.146) and *P. spelaea* (0.114–0.149); and it is higher than in jaguars (0.077–0.117), leopards (0.093–0.127), lions (0.098–0.137), *P. palaeosinensis* (0.124), and *P. atrox* (0.099–0.121), giving *P. zdanskyi* a massive cheek region, indicative of high bite forces (Supporting Information Fig. S3).

Mandibular morphology is similar to that of tigers in its straight ventral profile, and the mandible is heavily built. Mandible heights at four designated points (posterior to M_1_; at M_1_/P_4_; at P_4_/P_3_; anterior to P_3_) relative to mandible length are at the upper ranges of the corresponding ratios among other species of *Panthera*. It is traditionally considered that primitive tigers had proportionally smaller carnassials (P^4^ and M_1_) than those of modern tigers, and that tigers with relatively large carnassials first appear on the Asian mainland at Zhoukoudian in the Late Pleistocene [4], [15], [35], but *P. zdanskyi* demonstrates that this is incorrect. P^4^ length is 13.4% of CBL; this is at the upper range of the variation among tigers (0.104–0.141), jaguars (0.105–0.137), leopards (0.113–0.141), lions (0.111–0.142), and *P. spelaea* (0.102–0.137); and is higher than in *P. atrox* (0.106–0.125). M_1_ is 14.7% of mandible length, which is higher than among other species of *Panthera*. The relative sizes of P^3^, P_4_ and P_3_ are also at the upper end or even above the size ranges of those of other *Panthera* species, demonstrating that *P. zdanskyi* has very large teeth.

Most dental cusp proportions relative to overall crown length are fairly uniform among extant and extinct *Panthera* species with large overlaps in ratios. This is also the case for some of the teeth in the *P. zdanskyi*, for instance P^3^ metacone and paracone lengths, or P^4^ paracone length and width across the protocone. P^4^ has a distinct ectoparastyle, as in modern tigers, which is usually absent in other extant *Panthera* except its occasional presence in some jaguars. The P^4^ metastyle is relatively short (35.4% of crown length), which is below the ratio in *P. palaeosinensis* (0.385), but within the relative size ranges of other *Panthera* species. Interestingly the upper dentition in *P. zdanskyi* is more similar to that of tigers and also to other *Panthera* than the lower dentition (Supporting Information Fig. S4). M_1_ has a distinct talonid, and relatively very short paraconid and protoconid cusps (0.374 and 0.474 relative to M_1_ length, respectively), compared to the ratio ranges of tigers (0.370–0.433/0.456–0.575), jaguars (0.374–0.436/0.487–0.570), leopards (0.384–0.481/0.445–0.580), lions (0.358–0.447/0.489–0.596), *P. palaeosinensis* (0.639/0.631), *P. atrox* (0.387–0.472/0.477–0.583), and *P. spelaea* (0.406–0.461/0.464–0.504). The paraconid and protoconid are also very low in *P. zdanskyi* relative to M_1_ length (0.520 and 0.480, respectively), compared to those of other *Panthera* (0.6–0.8 paraconid height; and 0.52–0.67 protoconid height); other *Panthera* are quite similar for these ratio ranges. The length of the paraconid relative to P_4_ length in *P.zdanskyi* (0.178) is typical for other *Panthera*, but the protoconid is large (0.485), which is at the upper range for tigers (0.417–0.511), and is similar to those of lions, leopards, *P. atrox* and *P. spelaea*. *P. palaeosinensis* has a larger P_4_ paraconid and a much smaller protoconid than those of *P. zdanskyi*.

### Phylogenetic analyses

**Figure 3:**
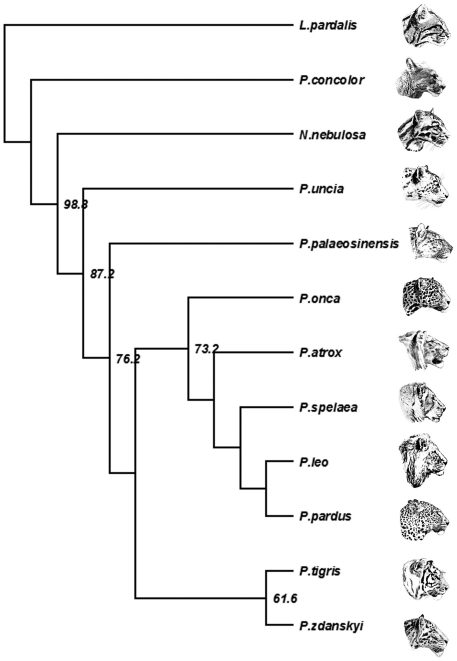
Strict consensus cladogram of two equally parsimonious trees of Pantherinae relationships (L = 103; CI = 0.66; HI = 0.34; RI = 0.65; RC = 0.43) based on 523 ingroup (*Neofelis*; *Panthera*) and 37 outgroup (*Leopardus pardalis*; *Puma concolor*) specimens from [**1**] computed in PAUP. *Panthera zdanskyi* is the sistertaxon of *P. tigris*. Bootstrap values indicated are 1000 replications. Art work by Velizar Simeonovski (Field Museum of Natural History, Chicago).

A cladistic analysis based on the database from [1] confirmed that *P. zdanskyi* is the sister taxon to the tiger (Fig. 3). In most characters, they are similar, except that *P. zdanskyi* has a small lacrimal process (in extant tigers it is large); the jugal-squamosal suture is positioned far posteriorly to the postorbital process; and M_1_ is relatively very large. The phylogenetic analysis confirms the very close (sister-group) relationship of *P. zdanskyi* and the tiger, but the character differences identify *P. zdanskyi* as a different species from the tiger. However, other subtle differences also indicate that *P. zdanskyi* cannot be grouped within the evolutionary radiation of the tiger, but should be regarded as a very closely related, separate species.

**Figure 4:**
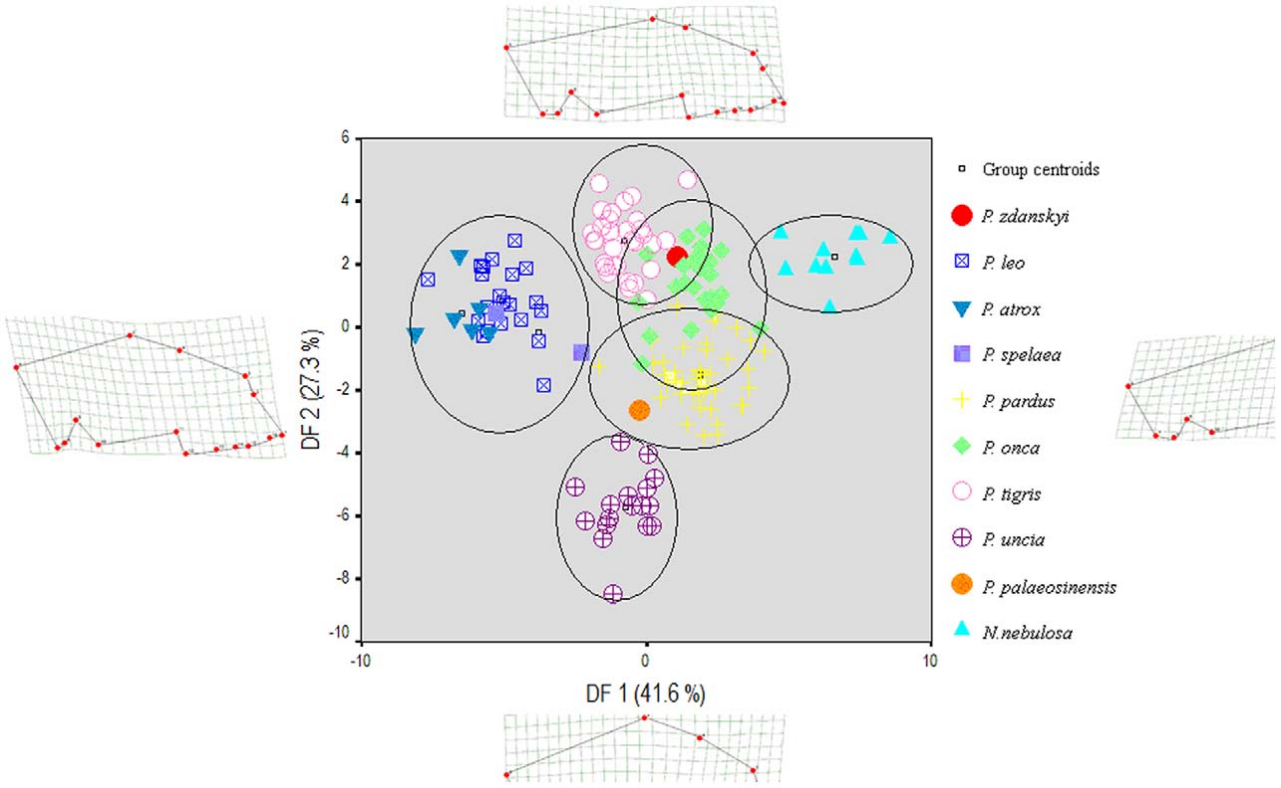
The shape of the cranium in *Panthera* spp. and *Neofelis nebulosa* analysed through a geometric morphometric thin plate splines analysis based on 16 landmarks, collectively capturing the overall shape of the cranium.

**Figure 5:**
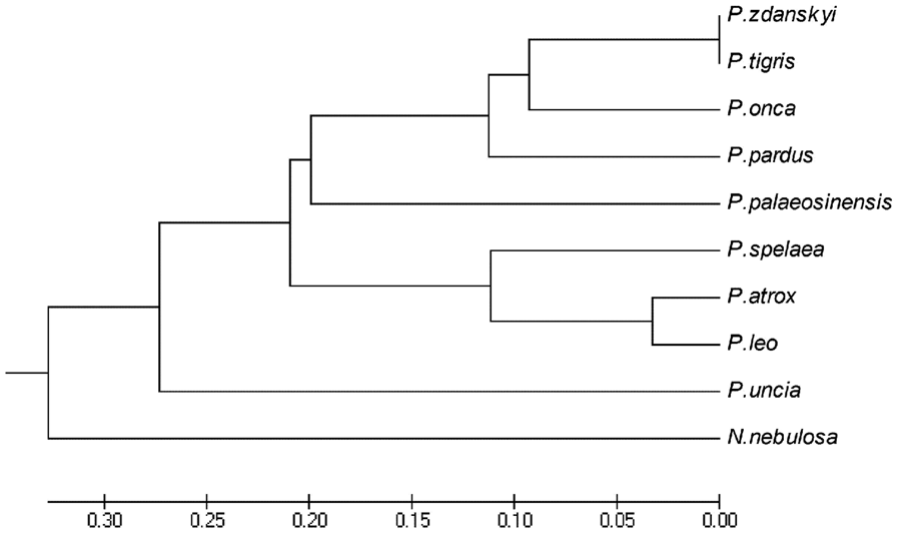
UPGMA distance-matrix tree constructed based on relative warp scores on a geometric morphometric analysis of cranial shape in the Pantherinae. The tree topology is broadly congruent with current knowledge on Pantherinae relationships based on parsimony analyses. *Panthera zdanskyi* is the sistertaxon to *P. tigris*, consistent with the tiger-like cranial morphology of *P. zdanskyi*.

A geometric morphometric analysis of the cranium showed that *P. zdanskyi* has a skull shape that is close to the cranial shape-space of modern tigers, but also tends towards that of jaguars, and although it groups within the morphospaces of both species, it is clearly most similar to that of extant tigers (Fig. 4). A Discriminant Function Analysis (DFA) was also performed on partial warps 1–13 and on the uniform warps X and Y, and a subsequent jack-knifed classification analysis also identified *P. zdanskyi* as a tiger. In contrast, the long-held tiger ancestor *P. palaeosinensis* is found to group well away from tigers and *P. zdanskyi*, and to fall within the morphospace of extant leopards. A UPGMA tree based on the distance-matrix derived from the cranial geometric morphometric analysis corroborated the phylogenetic study, showing that *P. zdanskyi* is the sister-taxon to the extant tiger (Fig. 5). *P. palaeosinensis*, however, has a more leopard-like cranial shape, congruent with other recent analyses [12].

**Figure 6:**
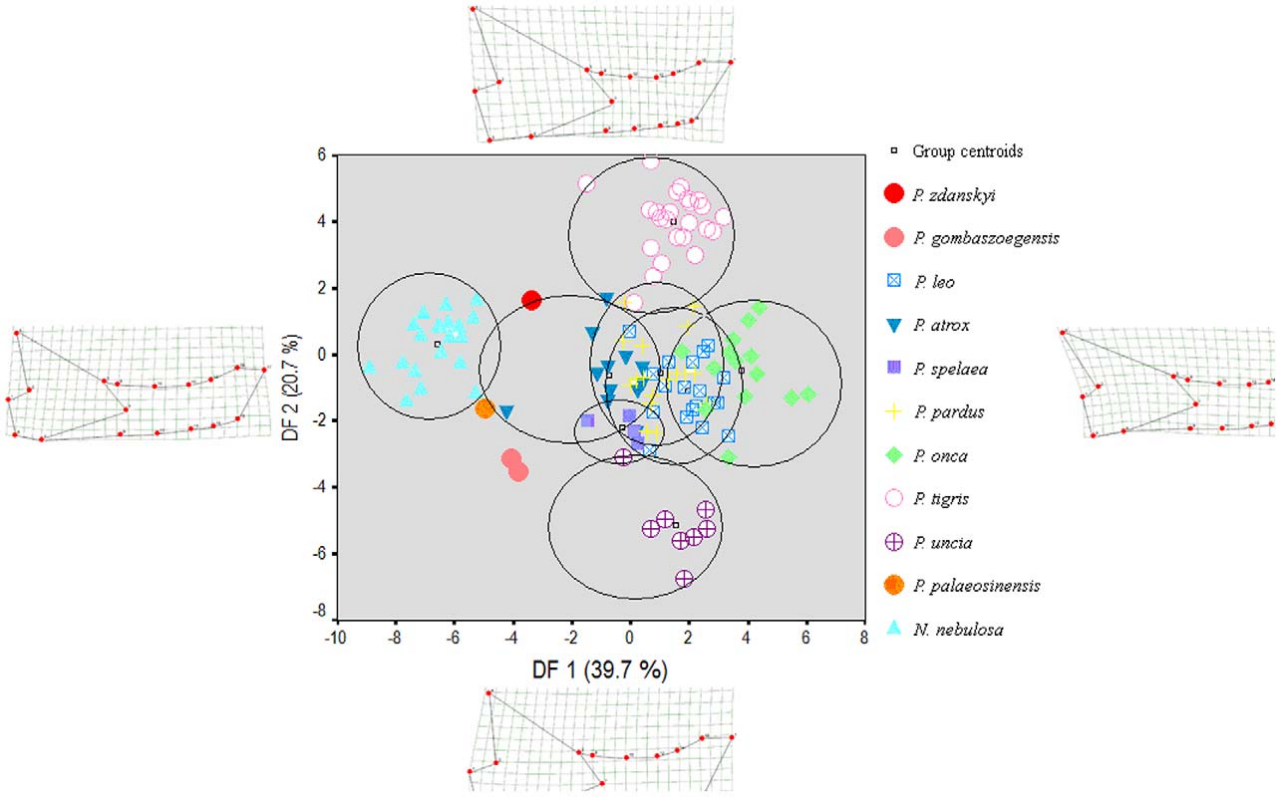
The shape of the mandible in *Panthera* spp. and *Neofelis nebulosa* analysed through a geometric morphometric thin plate splines analysis based on 18 landmarks, collectively capturing the overall shape of the mandible.

In contrast, a geometric morphometric analysis of the mandible shows that *P. zdanskyi* has a less tiger-like mandibular shape which does not fall within the morphospace of extant tigers (Fig. 6), and in some respects it appears to be intermediate between the morphospaces occupied by clouded leopards, tigers, and the great Late Pleistocene “jaglion” [34], *Panthera atrox*. *P. palaeosinensis* also has a rather leopard-like mandibular shape which is consistent with comparisons of cranial shape and dental characteristics. The differences in mandible shape between *P. zdanskyi* and *P. palaeosinensis* appear to be less than was the case for cranial shape.

In summary, cranio-dental morphology, shape analyses, and character distribution of *P. zdanskyi* corroboratively and unanimously indicate that it has a close affinity to the extant tiger and thus it firmly removes *P. palaeosinensis* as a potential ancestor of the tiger lineage. The combination of a tiger-like cranium and upper dentition, and a slightly less tiger-like mandible and lower dentition is indicative of a distinct species that is probably ancestral to the lineage leading to extant tiger diversity, as also indicated by character distribution and phylogenetic analysis. *P. zdanskyi* is the oldest known complete skull of a pantherine felid hitherto discovered, and it lends support to the notion that the tiger lineage originated in the earliest Pleistocene (traditionally considered Late Pliocene) in North-western China.

## Discussion

**Figure 7:**
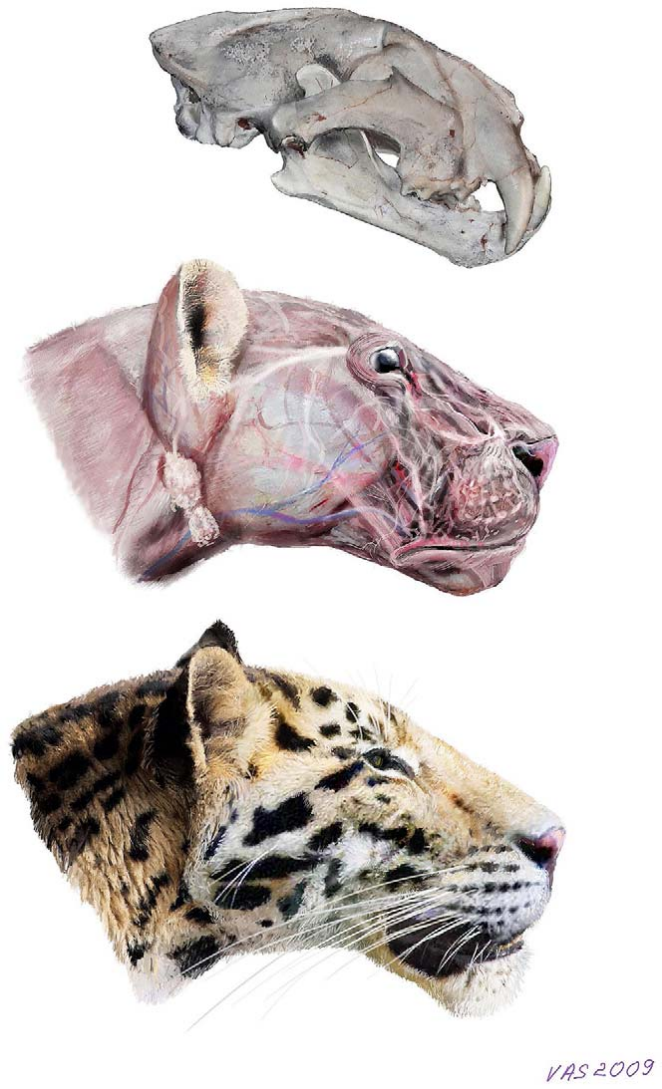
Artist's reconstruction of the Longdan tiger (*Panthera zdanskyi* sp. nov.), illustrated by Velizar Simeonovski (Field Museum of Natural History, Chicago). Myology reconstruction was done according to current knowledge of felid soft part anatomy, but coat morphology is tentative.

*Panthera zdanskyi* is an ancient, very primitive member of the particular *Panthera* species-lineage of which the extant tiger represents the crown taxon. In many ways it is morphologically surprisingly similar to extant tigers, given that it is more than two million years old, but distinct differences are also apparent. As such, it may not have shared the same coat morphology as extant tigers (Fig. 7). In light of the above, we propose an informal vernacular name for *Panthera zdanskyi*, the Longdan tiger.

In reconstructing the evolution of the tiger lineage, there are two principal aspects to consider. Firstly, the origin and divergence from other *Panthera*, species-lineages; and, secondly, the biogeographical history of the tiger species-lineage, including regional diversification. The biogeography of the tiger is fairly well known because of a good Middle-Late Pleistocene fossil record, but prior to the discovery of the Longdan tiger, fossils from the earliest Pleistocene were unknown. Accordingly, current knowledge of the divergence of the tiger lineage from other *Panthera* depends largely on molecular studies [9], [25], [36], [37]. Molecular data indicate that the radiation of modern felid lineages began with the divergence of the *Panthera* lineage around 10.8 MYA, and probably occurred in Southeast Asia. Soon afterwards, this was followed by a rapid radiation leading to the five extant *Panthera* species, among which the tiger and snow leopard, *P. uncia*, could share a sistergroup relationship [9]. The latter is, however, disputed by most morphological studies [1], [3], [38], [39, this study] and several molecular studies as well [40]–[42].

**Figure 8:**
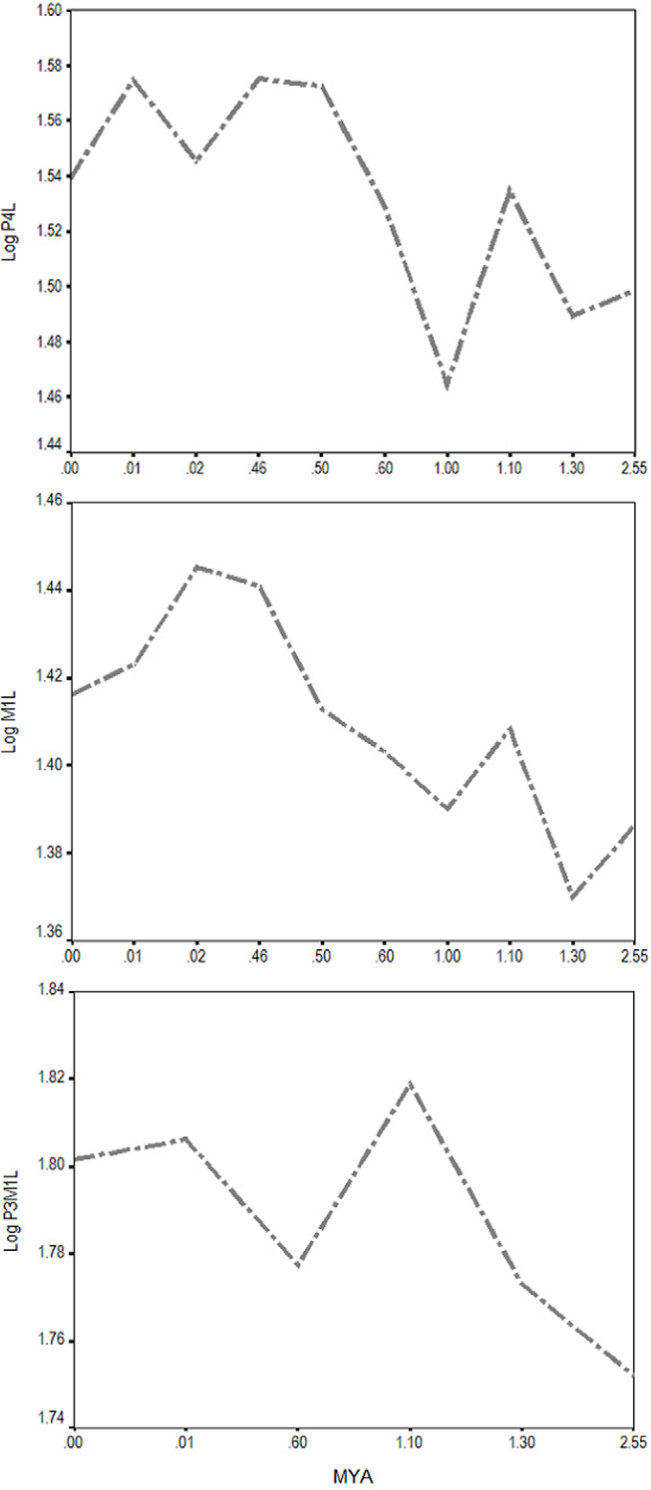
The size-change of tigers through the Late Pliocene-Pleistocene, using carnassial (P^4^ and M_1_) crown lengths and p3-M1 length. Sample localities are: Longdan (Gansu); Trinil (Java); Lantian (Shanxi); Liucheng (Guangxi); Wanxian (Sichuan); Fuming (Yunnan); Zhoukoudian (Beijing); Shandindong (Beijing).

The completeness of the Longdan tiger permits a more comprehensive hypothesis of tiger morpho-evolution than has hitherto been possible. Tigers were originally jaguar-sized (Supporting Information Fig. S2, Appendix S1) with very large teeth and a robust skull, and the tiger-like cranium and upper dentition were present from early on, whereas the mandible and lower dentition were more primitive and evolved at a faster rate during subsequent evolution of this lineage. A similar pattern of mosaic evolution is present in the cheetah lineage, and the primitive cheetah, *Acinonyx kurteni*, from the same region as *P. zdanskyi* has a cheetah-like cranium but a more primitive dentition [43], suggesting that this pattern may be common in felid evolution. A metric comparison of tiger dentitions from the earliest Pleistocene to the Holocene from various regions in East and Southeast Asia also suggest that a dominant trend in tiger evolution was increase in size, although the pattern is complicated and non-linear (Fig. 8).

**Figure 9:**
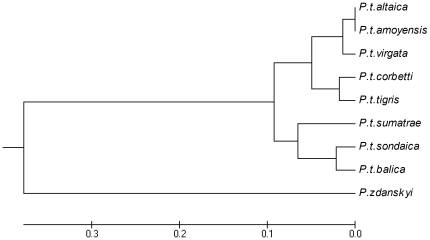
A UPGMA cluster analysis constructed from squared Euclidean distances derived from a Principal Components Analysis (PCA) on craniomandibular and dental proportions in putative tiger subspecies.

Compared to extant putative tiger subspecies, the skull of the Longdan tiger does not show any major differences other than size and minor differences in dental sizes and characteristics (Supporting Information Fig. S5). However, it is nonetheless clearly distinct from the modern tiger, as also shown above. Interestingly, multivariate Discriminant Analysis of size-adjusted cranio-mandibular and dental variables indicates that the Longdan tiger shows the greatest morphological affinity to extant and recently extinct Sunda Island tigers, and it appears to be less similar to the large Amur or Bengal tigers of the Asiatic mainland (Supporting Information Fig. S5, Fig. S6). However, it is morphologically clearly distinguishable from the skulls of all modern tigers (Fig. 9) and it is evidently not a part of the evolution leading to the intraspecific radiation of the extant tiger subspecies. This is perhaps not surprising giving that subspecies radiation is inferred to have occurred comparatively recently at less than 100 KYA [25], [26].

The UPGMA tree is broadly congruent with molecular studies on extant tiger subepecies, which have indicated a sister-group relationship between *P. t. altaica* and *P. t. virgata* [44], and with *P. t. corbetti* as the sister-group to these two [45], although not with *P. t amoyensis* and *P. t. tigris* included. *P. t. amoyensis* is often considered the most primitive extant tiger subspecies [44], [46]–[48], yet this is not replicated in the present study. The sister-group relationship of *P. t. corbetti* and *P. t. tigris* indicated here is congruent with earlier estimates of tiger phylogeny [48]–[50]. The sister-group relationship of *P. t. sondaica* and *P. t. balica* is also congruent with traditional views [50]–[55]. *P. t. sumatrae* is traditionally inferred to be distinct from other putative tiger subspecies genetically [44], [45], [56] and morphologically [50] and an earlier study on craniometric data indicated that it is more similar to *P. t. corbetti* than to other Sunda Island tigers [55], but in the current study it is found to group close to the other Sunda Island subspecies.

Expectedly, the Longdan tiger emerged as the most primitive tiger separated by a long distance from all extant tiger subspecies. This is congruent with an interpretation as an early branch of the tiger lineage but outside the evolutionary radiation within *P. tigris*. However, it is evident that even the earliest members of the tiger-lineage had already evolved an overall cranial morphology very similar to those of extant tigers, but the rates of evolution of the cranium, mandible and dentition have varied over the last ∼two million years. The overall skull morphology, inferred high bite forces, and the size and morphology of the dentition indicate that earliest Pleistocene tigers were already functionally and perhaps ecologically similar to modern tigers. Studies of extant tigers indicate that several factors have had marked influences on body size, including size and availability of prey, metabolic constraints on islands, and inter and intraspecific competition [57]. Tigers are dependent on large prey [58], [59] and cervids are the most important prey species across most of their geographic and faunistic range, but tigers also prey on wild pigs and bovines such as banteng and gaur [59]–[61]. The Pleistocene was a time of great adaptive radiations of cervids [62] and bovids [63], [64] in Southeast Asia, and assuming similar evolutionary constraints in the Early Pleistocene, the increase in tiger size may be an adaptation to increases in the size of their preferred prey.

## Materials and Methods

Morphological comparative material: Morphometric comparisons of *Panthera zdanskyi* with extant and extinct pantherines were performed using a database of skulls collected at museums across China, Europe, and the United States. We used a comparative database of 207 specimens of extant tigers of all putative subspecies; 207 lions; 66 jaguars; 100 leopards; and of extinct Pleistocene pantherines were used 14 specimens of *Panthera atrox*; 5 specimens of *P. spelaea*; two specimens of *P. gombaszoegensis*; and the holotype of *P. palaeosinensis*.

### Traditional morphometric analyses

We used bivariate comparative analyses (ANOVA and two-sample t-tests, as appropriate) and multivariate MANOVA, Principal Components Analysis (PCA), and stepwise Discriminant Function Analyses (DFA) on measured variables to compare the craniomandibular and dental morphology of *Panthera zdanskyi* to those of other pantherines.

### Geometric morphometric analyses

The morphology of the cranium and mandible of *Panthera zdanskyi* and its morphological resemblance to those of other pantherine species were also assessed using geometric morphometric analyses of the lateral aspect of the cranium and mandible. Geometric morphometric approaches study the shape of structures rather than covariance matrices and/or axes of dissimilarity, and, thus do not address linear distances among taxa by mathematical combinations of measured variables, as in traditional multivariate analyses; such approaches have the added advantage of separating morphological shape differences from differences resulting from size [65], [66]. We used the Thin Plate Splines (TPS) geometric morphometric function decomposed by its partial warps, which analyses shape deformations of structures compared to a predefined reference shape configuration [65], [66]. The TPS function
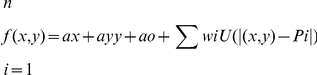
interpolates a surface which is fixed at the landmarks, and is computed so as to minimize the overall bending energy function
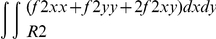
where bending energy is defined as the integral over R^2^ of the squares of the second derivatives; f_x_ and f_y_ are the separate thin-plate spline functions; w_i_ are coordinates; and U is r^2^log(r^2^), which is the so-called fundamental solution to the biharmonic equation ▵*^2^U* = 0. We digitized 16 landmarks at homologous points on the cranium and 18 landmarks on the mandible in tpsDig [67], collectively capturing the overall shape of the cranium and mandible, and performed relative warps analyses in tpsRelw [68]. At an α = 0, as used in this study, a relative warps analysis is a Principal Components Analysis of shape changes based on the covariance matrix of partial warp scores.

### Systematic analyses

The phylogenetic affinities of *Panthera zdanskyi* were assessed using a combination of cladistic parsimony analysis and distance-matrix analysis of cranial shape based on relative warp scores. We performed cladistic analyses using the maximum parsimony criterion and heuristic search in PAUP 4.0 [69] and bootstrap analyses (1000 replications) to assess the robustness and significance of the reported clades. The database used was from [1], [Supporting Information Appendix S2] , and comprised of 39 clouded leopards (22 *Neofelis diardi* and 17 *N. nebulosa*); 132 lions; 56 jaguars; 108 leopards; 120 tigers; 33 snow leopards; and 23 *P. atrox*; 10 *P. spelaea*; and the holotype of *P. palaeosinensis*; 24 pumas (*Puma concolor*) and 13 ocelots (*Leopardus pardalis*) were used as outgroups. We performed distance-matrix UPGMA (Unweighted Pair Group Method with Arithmetic mean) analyses in MEGA 4.1 [70], [71] on the relative warp scores [see 72 for method discussion] from a geometric morphometric analysis on cranial shape in pantherines. The use of this kind of distance-matrix approach for tree construction is not phenetics as traditionally understood because of the incorporation of an outgroup to provide an axis of polarity and a measure of derived similarity of shape coordinates.

## Supporting Information

Figure S1 A principal components analysis on size-adjusted metric variables of the anterior part of the upper dentition from the holotype and paratype of *P. zdanskyi* n. sp.; the holotype of *P. palaeosinensis*; the middle Pleistocene European jaguar (*P. gombaszoegensis*); the lower middle Pleistocene Chinese tigers (from Lantian) and a number of extant pantherines.
(DOC)

Figure S2 A comparison of condylobasal skull lengths (CBL) of 615 specimens of extant *Panthera* species, and the two fossil species, *Panthera palaeosinensis* and *P. zdanskyi* sp. nov. *P. zdanskyi* is similar in size to the smallest female specimens of modern tiger subspecies, but its morphology indicates that it was, in fact, a male, suggesting a size that is below even the smallest extant tiger males of any subspecies. Rather, *P. zdanskyi* appears to have been similar in size to jaguar males or large leopard males.
(DOC)

Figure S3 Bite forces were computed based on a model of relative, not absolute (i.e., in Newtons) force outputs from the temporalis and masseter muscles. Relative force output from the temporalis was computed as (((((ZW − ((BW+POW)/2))/2) * TFL)^0.5^) * MAT); and relative force output from the masseter was computed as ((((CFL + MSW)/2 * MSL)^0.5^) * MAM); where BW, is the width across the braincase; CFL, is the maximal anteroposterior length of the mandibular coronoid fossa; MAM, is the inlever moment arm of the masseter muscle from the mandibular cotyle to the ventral mandibular rim; MAT, is the inlever moment arm of the temporalis from the mandibular cotyle to the tip of the coronoid process; MSL, is the maximal anteroposterior length of the masseteric scar along the lateral face of the zygomatic arch; MSW, is the maximal dorsoventral width of the masseteric scar along the lateral face of the zygomatic arch; POW, is the width across the postorbital constriction of the skull; TFL, is the anteroposterior length of the temporal fossa in the skull from the posterior edge of the postorbital process to the anterior edge of the rim along the occipital crest; ZW, is the internal width across the zygomatic arches (i.e. not including the width or the arches themselves). This provides an estimate of the force outputs from the mandibular adductors along one side of the skull, and to get the estimated total force output the values were doubled.
(DOC)

Figure S4 Stepwise Discriminant Analyses of upper dentition variables (C^1^ crown length and alveolar width; P^3^ crown length and width; P^4^ crown length, width and length of the paracone and metastyle blades); and lower dentition variables (C_1_ crown height and alveolar width; P_3_ crown length; P_4_ crown length and width; and M_1_ crown length and width). For upper dentition, *Panthera tigris* ssp. are fossil tiger teeth from Lantian; and for lower dentition, *Panthera tigris* ssp. are fossil tiger teeth from Lantian and Yunnan. The analysis of upper dentition variables shows that *Panthera zdanskyi* groups close to extant and fossil tigers, whereas *P. palaeosinensis* groups closer to extant jaguars (*P. onca*) and Pleistocene jaguar-like cats (*P. gombaszoegensis*). In contrast to multivariate analyses on upper dentition, the analysis on lower dentition variables shows that *Panthera zdanskyi* groups intermediately between tigers and jaguars, and more closely to the latter. A jack-knifed classification analysis did, however, classify *P. zdanskyi* as a tiger rather than a jaguar. The morphological distinction between *P. zdanskyi* and *P. palaeosinensis* is less for lower dentition than for upper dentition.
(DOC)

Figure S5 3D plot of Principal Components (PC) 1–3 of a multivariate analysis on craniomandibular and dental proportions indicating that the Longdan tiger is distinctly different from all modern tigers on PC1, which is primarily related to its proportionally large teeth (in particular a well developed P^4^ protocone), and long tooth rows.
(DOC)

Figure S6 A plot of the first two Discriminant functions from a multivariate study Discriminant Function (DFA) study on Principal Component scores of craniomandibular and dental proportions in putative tiger subspecies without *a priori* classification. The Longdan tiger groups close to the group centroids of the extant Sunda island tiger subspecies, the Javan tiger (*Panthera tigris sondaica*); the Bali tiger (*P. t. balica*); and the Sumatra tiger (*P. t. sumatrae*).
(DOC)

Appendix S1 Actual body masses (kg) and condylobasal skull lengths (CBL; mm) in 19 specimens representing 6 species of extant pantherines used for computing regression analysis for predicting the body mass in *Panthera zdanskyi*. Regression analysis on species-averaged variables of Log_10_ CBL in millimetres and Log_10_ actual body mass in kg of 6 extant pantherines was used to predict the body mass of *P. zdanskyi*, and the result is 76.8 kg.
(DOC)

Appendix S2 Description of characters and data matrix used in phylogenetic analysis. For detail interpretation of character selection and coding, please see [1].
(DOC)

## References

[1] Christiansen P (2008). Phylogeny of the great cats (Felidae: Pantherinae), and the influence of fossil taxa and missing characters.. Cladistics.

[2] Werdelin L, O'Brien SJ, Johnson WE, Yamaguchi N, Macdonald DW, Loveridge AJ (2010). Phylogeny and evolution of cats (Felidae).. Biology and Conservation of Wild Felids.

[3] Hemmer H (1981). Die Evolution der Pantherkatzen. Modell zur Überprüfung der Brauchbarkeit der HENNIGschen Prinzipien der phylogenetischen Systematik für wirbeltier-paläontologische Studien.. Paläontol Z.

[4] Hemmer H, Tilson RL, Seal US (1987). The phylogeny of the tiger.. Tigers of the World. The Biology, Biopolitics, Management, and Conservation of an Endangered Species.

[5] Barry JC, Leakey MD, Harris JM (1987). Large carnivores (Canidae, Hyaenidae, Felidae) from Laetoli.. Laetoli – a Pliocene site in Tanzania.

[6] Turner A (1990). The evolution of the guild of larger terrestrial carnivores during the Plio-Pleistocene in Africa.. Geobios.

[7] Turner A, Antón M (1997). The big cats and their fossil relatives: An illustrated guide to their evolution and natural history.

[8] Werdelin L, Lewis ME (2005). Plio-Pleistocene Carnivora of eastern Africa: species richness and turnover patterns.. Zool J Linn Soc.

[9] Davis BW, Li G, Murphy WJ (2010). Supermatrix and species tree methods resolve phylogenetic relationships within the big cats, Panthera (Carnivora: Felidae).. Mol Phyl Evol.

[10] Zdansky O (1924). Jungtertiäre Carnivoren Chinas.. Palaeontol Sin (Ser C).

[11] Hemmer H (1967). Wohin gehört *“Felis” palaeosinensis* Zdansky, 1924, in systematischer Hinsicht?. N Jahrb Geol Paläontol Abh.

[12] Mazák JH (2010). What is *Panthera palaeosinensis*?. Mamm Rev.

[13] Pei WC (1934). On the Carnivora from locality 1 of Choukoutien.. Palaeontol Sin.

[14] Kitchener AC, Yamaguchi N, Tilson RL, Nyhus P (2010). What is a Tiger? Biogeography, morphology and taxonomy.. Tigers of the World. The science, politics and conservation of *Panthera tigris*.

[15] Brongersma LD (1935). Notes on some recent and fossil cats chiefly from the Malay Peninsula.. Zool Mededeel.

[16] Mazák V (1983). *Panthera tigris*.. Mamm Spec.

[17] Mazák V (1983). *Der Tiger*.. Neue Brehm-Bücherei.

[18] Kitchener AC, Dugmore AJ (2000). Biogeographical change in the tiger, *Panthera tigris*.. Anim Cons.

[19] Hooijer DA (1947). Pleistocene remains of *Panthera tigris* (Linnaeus) subspecies from Wanhsien, Szechwan, China, compared with fossil and recent tigers from other localities.. Am Mus Novitates.

[20] Guggisberg CAW (1975). Wild cats of the world.

[21] Herrington SJ, Tilson RL, Seal US (1987). Subspecies and the conservation of Panthera tigris: preserving genetic heterogeneity.. Tigers of the World. The biology, biopolitics, management, and conservation of an endangered species.

[22] Pocock RI (1929). Tigers.. J Bombay Nat Hist Soc.

[23] Mazák JH, Groves CP (2006). A taxonomic revision of the tigers of Southeast Asia.. Mamm Biol.

[24] Mazák JH (2008). Craniometric variation in the tiger (*Panthera tigris*): implications for patterns of diversity, taxonomy and conservation.. Mamm Biol.

[25] Luo S-J, Kim J-H, Johnson WE, van der Walt J, Martenson J, Yuhki N, Miquelle DG, Uphyrkina O, Goodrich JM, Quigley HB, Tilson R, Brady G, Martelli P, Subramaniam V, McDougal C, Hean S, Huang S-Q, Pan W, Karanth UK, Sunquist M, Smith JLD, O'Brien SJ (2004). Phylogeography and genetic ancestry of tigers (*Panthera tigris*).. PLoS Biol.

[26] Driscoll CA, Yamaguchi N, Bar-Gal GK, Roca AL, Luo S, MacDonald DW, O'Brien SJ (2009). Mitochondrial phylogeography illuminates the origin of the extinct Caspian tiger and its relationships to the Amur tiger.. PloS One.

[27] Qiu ZX, Deng T, Wang BY (2004). Early Pleistocene fauna from Longdan, Donxiang, Gansu, China.. Palaeontol Sin (NS C).

[28] Gradstein FM, Ogg JG, Smith AG, Bleeker W, Lourens LJ (2004). A new Geologic Time Scale, with special reference to Precambrian and Neogene.. Episodes.

[29] Gibbard P, van Kolfschoten T, Gradstein FM, Ogg JG, Smith AG (2004). The Pleistocene and Holocene Epochs.. A Geologic Time Scale.

[30] International Commission on Stratigraphy website http://www.stratigraphy.org/.

[31] Martin LD, Babiarz JP, Naples VL, Hearst J (2000). Three ways to be a saber-toothed cat.. Naturwiss.

[32] Mazák JH (2004). On the sexual dimorphism in the skull of the tiger (*Panthera tigris*).. Mamm Biol.

[33] Christiansen P (2008). Distinguishing skulls of lions (*Panthera leo*) and tigers (*Panthera tigris*).. Mamm Biol.

[34] Christiansen P, Harris JM (2009). Craniomandibular morphology and phylogenetic affinities of *Panthera atrox*: implications for the evolution and paleobiology of the lion lineage.. J Vert Paleontol.

[35] Hemmer H (1971). Fossil mammals of Java. II. Zur Fossilgeschichte des Tigers (*Panthera tigris* (L.) in Java.. Koninkl Nederlandse Akad Wetenschap (Ser B).

[36] Johnson WE, Eizirik E, Pscon-Slattery J, Murphy WJ, Antunes A, Teeling E, O'Brien SJ (2006). The Late Miocene radiation of modern Felidae: A genetic assessment.. Science.

[37] Luo S-J, Kim J-H, Johnson WE, Miquelle DG, Huang S-Q, Pan W-S, Smith JLD, O'Brien SJ (2006). Proceedings in phylogeography and genetic ancestry of tigers (*Panthera tigris*) in China and across their range.. Zool Res.

[38] Salles LO (1992). Felid phylogenetics: Extant taxa and skull morphology (Felidae, Aeluroidea).. Am Mus Novitates.

[39] Mattern MY, McLennan DA (2000). Phylogeny and speciation of felids.. Cladistics.

[40] O'Brien SJ, Collier GE, Benveniste RE, Nash WG, Newman AK, Simonson JM, Eichelberger MA, Seal US, Janssen D, Bush M, Wildt DE, Tilson RL, Seal US (1987). Setting the molecular clock in the Felidae: the great cats, *Panthera*.. Tigers of the World. The biology, biopolitics, management, and conservation of an endangered species.

[41] Johnson WE, O'Brien SJ (1997). Phylogenetic reconstruction of the Felidae using 16S rRNA and NADH-5 mitchondrial genes.. J Mol Evol.

[42] Yu L, Zhang Y-p (2005). Phylogenetic studies of pantherine cats (Felidae) based on multiple genes, with novel application of nuclear β-fibrinogen intron 7 to carnivores.. Mol Phyl Evol.

[43] Christiansen P, Mazak J (2009). A primitive Late Pliocene cheetah and evolution of the cheetah lineage.. Proc Natl Acad Sci.

[44] Driscoll CA, Yamaguchi N, Bar-Gal GK, Roca AL, Luo S, MacDonald DW, O'Brien SJ (2009). Mitochondrial phylogeography illuminates the origin of the extinct Caspian tiger and its relationships to the Amur tiger.. PloS One.

[45] Luo S-J, Kim J-H, Johnson WE, van der Walt J, Martenson J, Yuhki N, Miquelle DG, Uphyrkina O, Goodrich JM, Quigley HB, Tilson R, Brady G, Martelli P, Subramaniam V, McDougal C, Hean S, Huang S-Q, Pan W, Karanth UK, Sunquist M, Smith JLD, O'Brien SJ (2004). Phylogeography and genetic ancestry of tigers (*Panthera tigris*).. PLoS Biol.

[46] Hemmer H (1981). Die Evolution der Pantherkatzen. Modell zur Überprüfung der Brauchbarkeit der HENNIGschen Prinzipien der phylogenetischen Systematik für wirbeltier-paläontologische Studien.. Paläontol Z.

[47] Hemmer H, Tilson RL, Seal US (1987). The phylogeny of the tiger.. Tigers of the World. The Biology, Biopolitics, Management, and Conservation of an Endangered Species.

[48] Herrington SJ, Tilson RL, Seal US (1987). Subspecies and the conservation of Panthera tigris: preserving genetic heterogeneity.. Tigers of the World. The biology, biopolitics, management, and conservation of an endangered species.

[49] Mazák V (1968). Nouvelle sous-espece de tigre provenant de L'Asie du Sud-Est.. Mammalia.

[50] Mazák V (1983). *Der Tiger*.. Neue Brehm-Bücherei.

[51] Schwarz E (1912). Notes on Malay tigers, with description of a new form from Bali.. Ann Mag Nat Hist.

[52] Schwarz E (1913). Der Bali-Tiger.. Ber Senckenberg Naturforsch Ges.

[53] Sody HJV (1932). The balinese tiger *Panthera tigris balica* (Schwarz).. J Bombay Nat Hist Soc.

[54] Seidensticker J, Tilson RL, Seal US (1987). Bearing witness: Observations on the extinction of *Panthera tigris balica* and *Panthera tigris sondaica*.. Tigers of the World. The Biology, Biopolitics, Management, and Conservation of an Endangered Species.

[55] Mazák JH, Groves CP (2006). A taxonomic revision of the tigers (*Panthera tigris*) of Southeast Asia.. Mamm Biol.

[56] Cracraft J, Felsenstein J, Vaughn J, Helm-Bychowski K (1998). Sorting out tigers (*Panthera tigris*): mitochondrial sequences, nuclear inserts, systematics and conservation genetics.. Anim Cons.

[57] Kitchener AC, Seidensticker J, Christie S, Jackson P (1999). Tiger distribution, phenotypic variation and conservation issues.. Riding the tiger. Tiger conservation in human-dominated landscapes.

[58] Karanth KU, Sunquist ME (2008). Behavioural correlates of predation by tiger (*Panthera tigris*), leopard (*Panthera pardus*), and dhole (*Cuon alpinus*) in Nagarahole, India.. J Zool Lond.

[59] Andheria AP, Karanth KU, Kumar NS (2007). Diet and prey profiles of three sympatric large carnivores in Bandipur Tiger Reserve, India.. J Zool Lond.

[60] Hoogewerf A (1970). Udjung Kulon. The land of the last Javan rhinoceros, with local and general data on the most important faunal species and their preservation in Indonesia.

[61] Sunquist M, Karanth KU, Sunquist F, Seidensticker J, Christie S, Jackson P (1999). Ecology, behaviour and resilience of the tiger and its conservation needs.. Riding the tiger. Tiger conservation in human-dominated landscapes.

[62] Geist V (1983). On the evolution of the Ice Age mammals and its significance to an understanding of speciations.. Assoc S-E Biol Bull.

[63] Kurtén B (1971). The Age of Mammals.

[64] MacEachern S, McEwn J, Goddard M (2009). Phylogenetic reconstruction and the identification of ancient polymorphism in the Bovini tribe (Bovidae, Bovinae).. BMC Genom.

[65] Bookstein FL (1991). Morphometric Tools for Landmark Analysis: Geometry and Biology.

[66] Zelditch ML, Swiderski DL, Sheets HD, Fink WL (2004). Geometric morphometrics for biologists. A primer.

[67] Rohlf FJ (2004). tpsDig, digitize landmarks and outlines, ver. 2.0..

[68] Rohlf FJ (2004). tpsRelw, ver. 1.39..

[69] Swofford DL (1998). PAUP*. Phylogenetic Analysis Using Parsimony (*and other methods). Ver. 4.

[70] Tamura K, Dudley J, Nei M, Kumar S (2007). MEGA4: Molecular Evolutionary Genetics Analysis (MEGA software version 4.0.. Mol Biol Evol.

[71] Kumar S, Nei M, Dudley J, Tamura K (2008). MEGA: A biologist-centric software for evolutionary analysis of DNA and protein sequences.. Brief Bioinformat.

[72] Cole TM, Lele S, Richtsmeier JT, MacLeod N, Forey PL (2002). A parametric bootstrap approach to the detection of phylogenetic signals in landmark data.. Morphology, shape and phylogeny.

