# Correction: Oldest Known Pantherine Skull and Evolution of the Tiger

**DOI:** 10.1371/annotation/a60b7ac3-7f06-465b-a8df-2f359d59a021

**Published:** 2012-01-18

**Authors:** Ji H. Mazák, Per Christiansen, Andrew C. Kitchener

The electronic version of this document does not represent a published work according to the International Code of Zoological Nomenclature (ICZN), and hence the nomenclatural acts contained in the electronic version are not available under that Code from the electronic edition. Therefore, a separate edition of this document was produced by a method that assures numerous identical and durable copies, and those copies were simultaneously obtainable for the purpose of providing a public and permanent scientific record, in accordance with Article 8.1 of the Code on December 14th, 2011. The separate print-only edition is available on request from PLoS by sending a request to PLoS ONE, 1160 Battery Street, Suite 100, San Francisco, CA 94111, USA along with a check for $10 (to cover printing and postage) payable to "Public Library of Science".

In addition, this published work and the nomenclatural acts it contains have been registered in ZooBank, the proposed online registration system for the ICZN. The ZooBank LSIDs (Life Science Identifiers) can be resolved and the associated information viewed through any standard web browser by appending the LSID to the prefix "http://zoobank.org/". The LSID for this publication is: (urn:lsid:zoobank.org:act:A7A75025-5E17-4CAA-B6FE-400301F8A57D).

