# Supplementary material for: Oldest Known Pantherine Skull and Evolution of the Tiger
Source: PLoS One. 2011 Oct 10;6(10):e25483. doi: 10.1371/journal.pone.0025483 (PMC3189913; doi:10.1371/journal.pone.0025483)
Supplement: Figure S1 — A principal components analysis on size-adjusted metric variables of the anterior part of the upper dentition from the holotype and paratype of P. zdanskyi n. sp.; the holotype of P. palaeosinensis; the middle Pleistocene European jaguar (P. gombaszoegensis); the lower middle Pleistocene Chinese tigers (from Lantian) and a number of extant pantherines. (DOC) [file pone.0025483.s001.doc]

**Figure S1**. A Principal Components Comparison on holotype and paratype of *Panthera zdanskyi* sp. nov. to other extant and extinct pantherines.


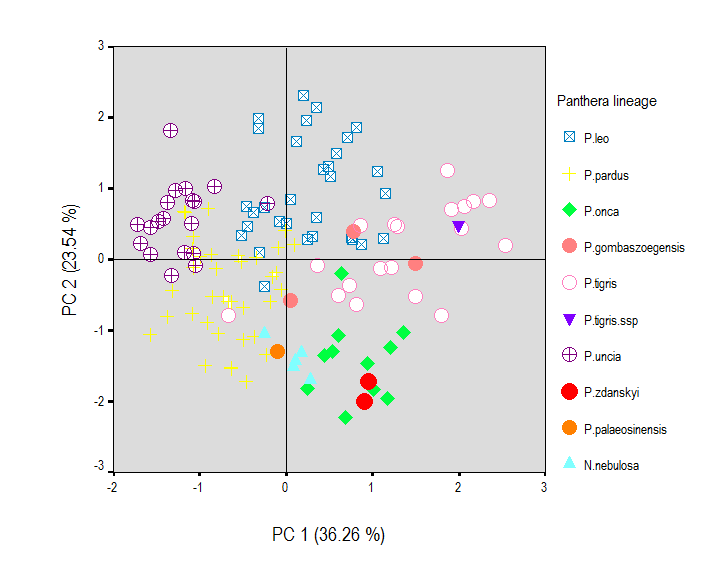


The comparisons were made based on the anterior part of the upper dentition (C [LC, WC]; P^3^ [LP3, WP3], and anterior part of P^4^ [LP4, WP4]). Even when using only this limited material, it is evident that the holotype and paratype of *Panthera zdanskyi* are nearly identical, and neither group close to *P. palaeosinensis*.
