# Supplementary material for: Oldest Known Pantherine Skull and Evolution of the Tiger
Source: PLoS One. 2011 Oct 10;6(10):e25483. doi: 10.1371/journal.pone.0025483 (PMC3189913; doi:10.1371/journal.pone.0025483)
Supplement: Figure S2 — A comparison of condylobasal skull lengths (CBL) of 615 specimens of extant Panthera species, and the two fossil species, Panthera palaeosinensis and P. zdanskyi sp. nov. P. zdanskyi is similar in size to the smallest female specimens of modern tiger subspecies, but its morphology indicates that it was, in fact, a male, suggesting a size that is below even the smallest extant tiger males of any subspecies. Rather, P. zdanskyi appears to have been similar in size to jaguar males or large leopard males. (DOC) [file pone.0025483.s002.doc]

**Figure S2**. Cranial size in *Panthera zdanskyi* and extant *Panthera*.


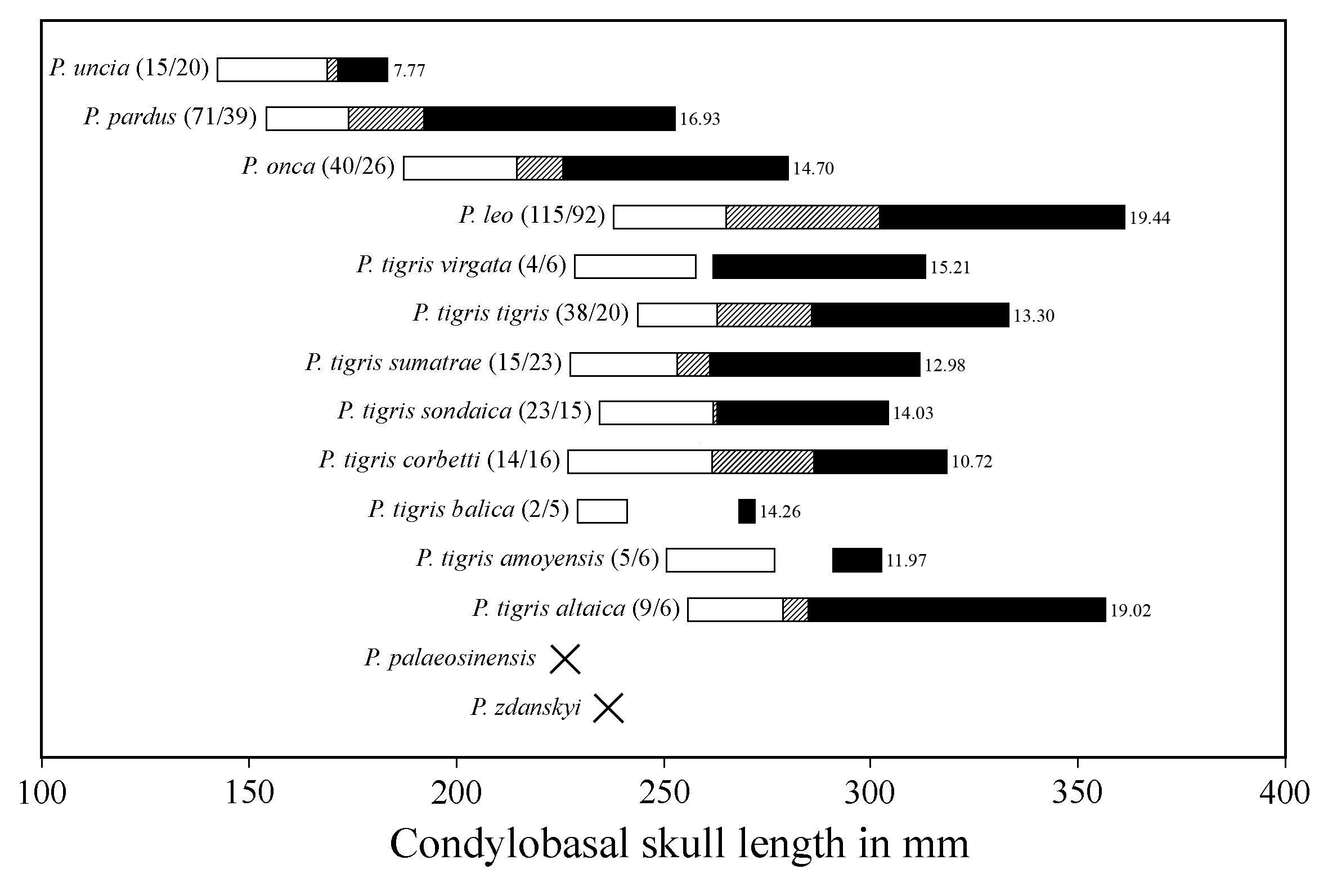


A comparison of condylobasal skull lengths (CBL) in 615 specimens of extant *Panthera* species, and the two fossil species, *Panthera palaeosinensis* and *P. zdanskyi* sp. nov. *P. zdanskyi* is similar in size to the smallest female specimens of modern tiger subspecies, but its morphology indicates that it was, in fact, a male, suggesting a size that is outside even the smallest extant tiger males of any subspecies. Rather, *P. zdanskyi* appears to have been similar in size to jaguar males or large leopard males. The numbers after each taxon name are number of males/number of females; and the numbers after the bars indicate the degree of sexual dimorphism in CBL sizes. White bars indicate female size distribution; black bars indicate male size distribution; and overlaps between the sexes are indicated by cross-hatching.
