# Supplementary material for: Oldest Known Pantherine Skull and Evolution of the Tiger
Source: PLoS One. 2011 Oct 10;6(10):e25483. doi: 10.1371/journal.pone.0025483 (PMC3189913; doi:10.1371/journal.pone.0025483)
Supplement: Figure S3 — Bite forces were computed based on a model of relative, not absolute (i.e., in Newtons) force outputs from the temporalis and masseter muscles. Relative force output from the temporalis was computed as (((((ZW − ((BW+POW)/2))/2) * TFL)0.5) * MAT); and relative force output from the masseter was computed as ((((CFL + MSW)/2 * MSL)0.5) * MAM); where BW, is the width across the braincase; CFL, is the maximal anteroposterior length of the mandibular coronoid fossa; MAM, is the inlever moment arm of the masseter muscle from the mandibular cotyle to the ventral mandibular rim; MAT, is the inlever moment arm of the temporalis from the mandibular cotyle to the tip of the coronoid process; MSL, is the maximal anteroposterior length of the masseteric scar along the lateral face of the zygomatic arch; MSW, is the maximal dorsoventral width of the masseteric scar along the lateral face of the zygomatic arch; POW, is the width across the postorbital constriction of the skull; TFL, is the anteroposterior length of the temporal fossa in the skull from the posterior edge of the postorbital process to the anterior edge of the rim along the occipital crest; ZW, is the internal width across the zygomatic arches (i.e. not including the width or the arches themselves). This provides an estimate of the force outputs from the mandibular adductors along one side of the skull, and to get the estimated total force output the values were doubled. (DOC) [file pone.0025483.s003.doc]

**Figure S3**. Bite force estimation.

Bite forces were computed based on a model of relative, not absolute (i.e., in Newtons) force outputs from the temporalis and masseter muscles. Relative force output from the temporalis was computed as (((((ZW - ((BW+POW)/2))/2) * TFL)^0.5^) * MAT); and relative force output from the masseter was computed as ((((CFL + MSW)/2 * MSL)^0.5^) * MAM); where BW, is the width across the braincase; CFL, is the maximal anteroposterior length of the mandibular coronoid fossa; MAM, is the inlever moment arm of the masseter muscle from the mandibular cotyle to the ventral mandibular rim; MAT, is the inlever moment arm of the temporalis from the mandibular cotyle to the tip of the coronoid process; MSL, is the maximal anteroposterior length of the masseteric scar along the lateral face of the zygomatic arch; MSW, is the maximal dorsoventral width of the masseteric scar along the lateral face of the zygomatic arch; POW, is the width across the postorbital constriction of the skull; TFL, is the anteroposterior length of the temporal fossa in the skull from the posterior edge of the postorbital process to the anterior edge of the rim along the occipital crest; ZW, is the internal width across the zygomatic arches (i.e. not including the width or the arches themselves). This provides an estimate of the force outputs from the mandibular adductors along one side of the skull, and to get the estimated total force output the values were doubled.


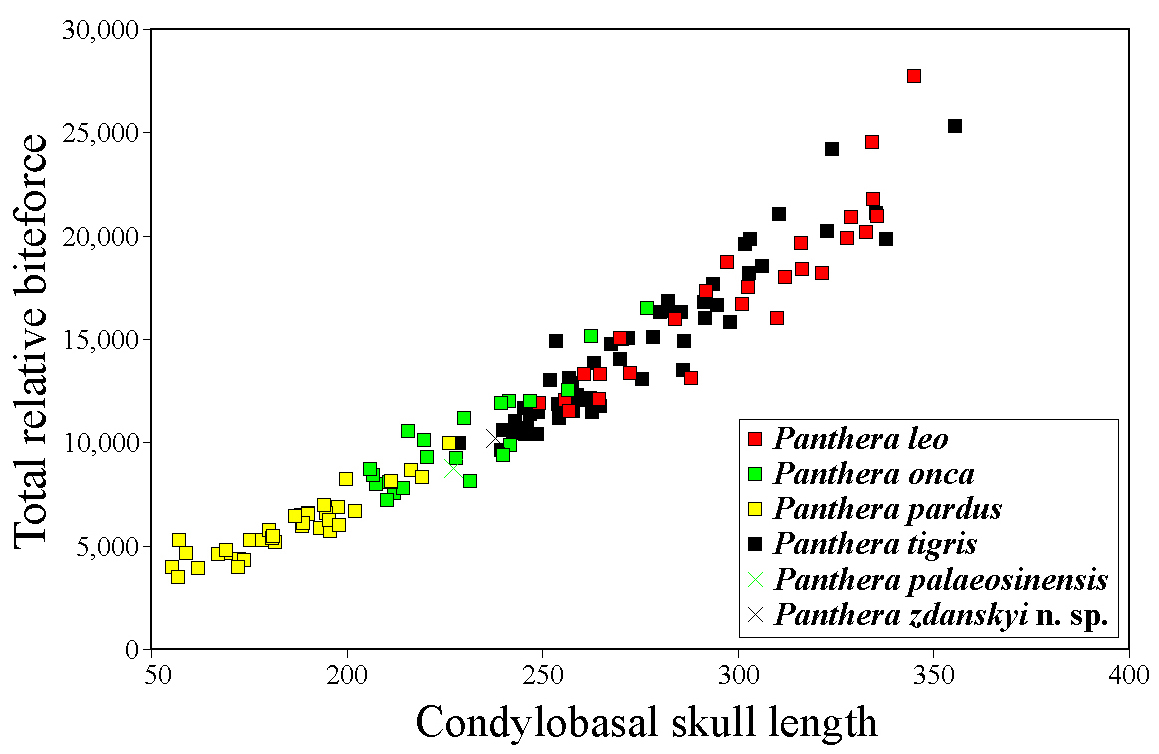


*Panthera zdanskyi* is estimated to have had a high relative bite force, typical of extant tigers and jaguars, and congruent with the robust nature of the cranium and mandible, and the large sagittal crest.
