# Supplementary material for: Oldest Known Pantherine Skull and Evolution of the Tiger
Source: PLoS One. 2011 Oct 10;6(10):e25483. doi: 10.1371/journal.pone.0025483 (PMC3189913; doi:10.1371/journal.pone.0025483)
Supplement: Figure S4 — Stepwise Discriminant Analyses of upper dentition variables (C1 crown length and alveolar width; P3 crown length and width; P4 crown length, width and length of the paracone and metastyle blades); and lower dentition variables (C1 crown height and alveolar width; P3 crown length; P4 crown length and width; and M1 crown length and width). For upper dentition, Panthera tigris ssp. are fossil tiger teeth from Lantian; and for lower dentition, Panthera tigris ssp. are fossil tiger teeth from Lantian and Yunnan. The analysis of upper dentition variables shows that Panthera zdanskyi groups close to extant and fossil tigers, whereas P. palaeosinensis groups closer to extant jaguars (P. onca) and Pleistocene jaguar-like cats (P. gombaszoegensis). In contrast to multivariate analyses on upper dentition, the analysis on lower dentition variables shows that Panthera zdanskyi groups intermediately between tigers and jaguars, and more closely to the latter. A jack-knifed classification analysis did, however, classify P. zdanskyi as a tiger rather than a jaguar. The morphological distinction between P. zdanskyi and P. palaeosinensis is less for lower dentition than for upper dentition. (DOC) [file pone.0025483.s004.doc]

**Figure S4**. Multivariate comparison of upper dentitions among *Panthera*.


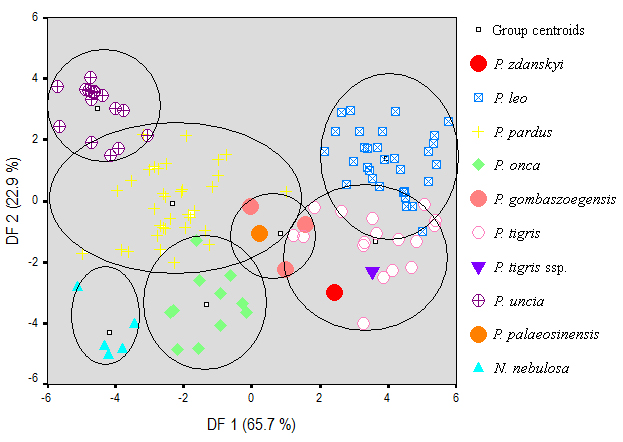


Stepwise Discriminant Analyses of upper dentition variables (C^1^ crown length and alveolar width; P^3^ crown length and width; P^4^ crown length, width and length of the paracone and metastyle blades). *Panthera tigris* ssp. are fossil tiger teeth from Lantian. The analysis shows that *Panthera zdanskyi* groups close to extant and fossil tigers, whereas *P. palaeosinensis* groups closer to extant jaguars (*P. onca*) and Pleistocene jaguar-like cats (*P. gombaszoegensis*).
