# Supplementary material for: Oldest Known Pantherine Skull and Evolution of the Tiger
Source: PLoS One. 2011 Oct 10;6(10):e25483. doi: 10.1371/journal.pone.0025483 (PMC3189913; doi:10.1371/journal.pone.0025483)
Supplement: Figure S5 — 3D plot of Principal Components (PC) 1–3 of a multivariate analysis on craniomandibular and dental proportions indicating that the Longdan tiger is distinctly different from all modern tigers on PC1, which is primarily related to its proportionally large teeth (in particular a well developed P4 protocone), and long tooth rows. (DOC) [file pone.0025483.s005.doc]

**Figure S5**. Principal Components Analysis of craniomandibular and dental proportions in tiger subspecies.

3D plots of Principal Components (PC) 1-4 from a multivariate analysis on craniomandibular and dental proportions, indicating that the Longdan tiger is distinctly different from all modern tigers on PC1, which is primarily related to its proportionally large teeth (in particular a well developed P^4^ protocone), and long tooth rows. PC2 is able to separate most modern putative tiger subspecies into two major clusters: one contains Indochina, India and the Sunda Islands; the other contains Amur, Caspian, South China, and Sumatra. The major differences lie in the former having proportionally long tooth rows, a more elongate cranium, and a relatively long distance between the infraorbitial foramina; whereas the latter have a proportionally tall coronoid process; tall occiput, shorter nasals and P^4^ with a well developed protocone.


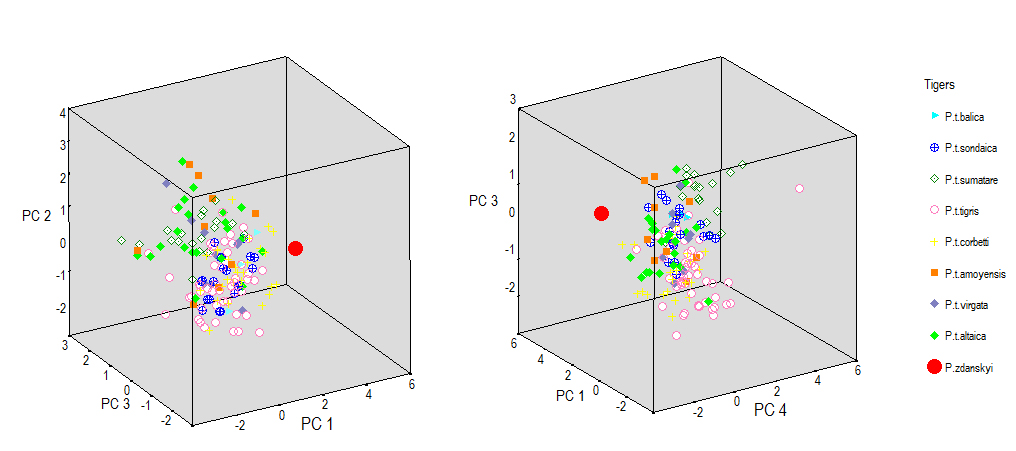


The 3D plot of PC’s 1, 3, 4, indicate that the Sumatran tigers are separated from India/Indochina and Java in having proportionally widened muzzle and short nasal. This separation is congruent with molecular studies [1].

1. Cracraft J, Felsenstein J, Vaughn J, Helm-Bychowski K (1998) Sorting out tigers (*Panthera tigris*): mitochondrial sequences, nuclear inserts, systematics and conservation genetics. Anim Cons 1: 139-150.
