# Supplementary material for: Oldest Known Pantherine Skull and Evolution of the Tiger
Source: PLoS One. 2011 Oct 10;6(10):e25483. doi: 10.1371/journal.pone.0025483 (PMC3189913; doi:10.1371/journal.pone.0025483)
Supplement: Figure S6 — A plot of the first two Discriminant functions from a multivariate study Discriminant Function (DFA) study on Principal Component scores of craniomandibular and dental proportions in putative tiger subspecies without a priori classification. The Longdan tiger groups close to the group centroids of the extant Sunda island tiger subspecies, the Javan tiger (Panthera tigris sondaica); the Bali tiger (P. t. balica); and the Sumatra tiger (P. t. sumatrae). (DOC) [file pone.0025483.s006.doc]

**Figure S6**. Discriminant Function Analysis on Principal Components from a study of craniomandibular and dental proportions in tiger subspecies.

A plot of the first two discriminant functions from a multivariate Discriminant Function Analysis (DFA) study on Principal Component scores of craniomandibular and dental proportions in putative tiger subspecies without *a priori* classification. The Longdan tiger groups close to the group centroids of the extant (or rather, recently extinct in the case of Java and Bali tigers [1, 2]) Sunda island tiger subspecies, the Javan tiger (*Panthera tigris sondaica*); the Bali tiger (*P. t. balica*); and the Sumatra tiger (*P. t. Sumatrae*). However, the DFA plot also indicates that there is significant overlap between several of the putative tiger subspecies.


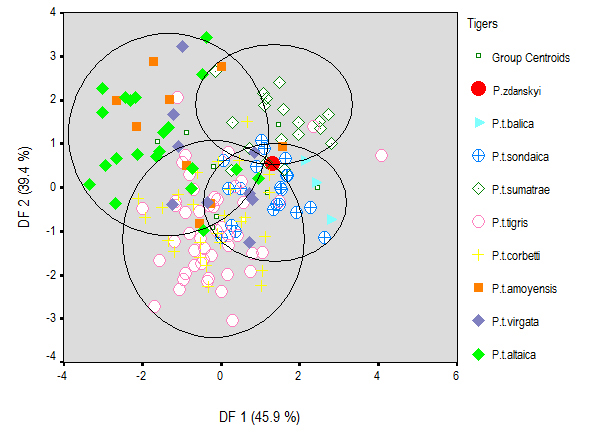


1. Seidensticker J (1987) Bearing witness: Observations on the extinction of *Panthera tigris balica* and *Panthera tigris sondaica*. In: Tilson RL, Seal US, editors. Tigers of the World. The Biology, Biopolitics, Management, and Conservation of an Endangered Species. New Jersey: Noyes Publ. pp. 1-8.

2. Ashraf MA (2006) The extirpation of Bali and Javan tiger: lessons from the past. Tigerpaper 33: 3-8.
