# Supplementary material for: Oldest Known Pantherine Skull and Evolution of the Tiger
Source: PLoS One. 2011 Oct 10;6(10):e25483. doi: 10.1371/journal.pone.0025483 (PMC3189913; doi:10.1371/journal.pone.0025483)
Supplement: Appendix S1 — Actual body masses (kg) and condylobasal skull lengths (CBL; mm) in 19 specimens representing 6 species of extant pantherines used for computing regression analysis for predicting the body mass in Panthera zdanskyi. Regression analysis on species-averaged variables of Log10 CBL in millimetres and Log10 actual body mass in kg of 6 extant pantherines was used to predict the body mass of P. zdanskyi, and the result is 76.8 kg. (DOC) [file pone.0025483.s007.doc]

**Appendix 1**. Estimation of body mass in *Panthera zdanskyi*.

Actual body masses (kg) and condylobasal skull lengths (CBL; mm) in 19 specimens representing 6 species of extant pantherines used for computing regression analysis for predicting the body mass in *Panthera zdanskyi*. Institutional abbreviations: BM, Natural History Museum, London; CN, Zoological Museum, Copenhagen.

BM CBL Taxon

19.1 154.1 *Neofelis nebulosa* BM47.685 (♂)

150.0 290.1 *Panthera leo* CN7364 (♀)

170.0 325.0 *Panthera leo* CN6043 (♂)

203.0 359.7 *Panthera leo* CN7231 (♂)

47.3 219.8 *Panthera onca* CN5659 (♀)

67.4 229.7 *Panthera onca* CN5660 (♂)

51.5 222.4 *Panthera onca* CN5707 (♀)

71.0 246.6 *Panthera onca* CN6221 (♂)

56.7 188.2 *Panthera pardus* CN5661 (♀)

57.9 210.1 *Panthera pardus* CN5662 (♂)

44.5 199.7 *Panthera pardus* CN8462 (♂)

145.0 302.3 *Panthera tigris* CN5668 (♂)

115.0 261.6 *Panthera tigris* CN5669 (♂)

221.0 334.2 *Panthera tigris* CN5697 (♂)

230.0 350.9 *Panthera tigris* CN5698 (♂)

225.0 337.8 *Panthera tigris* CN6049 (♂)

39.5 171.7 *Panthera uncia* CN5216 (♀)

34.0 166.3 *Panthera uncia* CN6046 (♀)

43.1 182.2 *Panthera uncia* CN6047 (♂)

Regression analysis on species-averaged variables of Log_10_ CBL in millimetres and Log_10_ actual body mass in kg of 6 extant pantherines. CBL is the dependent variable Y

a±95%CI b±95CI r F p

1.723±0.158 0.345±0.087 0.984 122.597 <0.001

With a CBL of 236.3 mm, the holotype of *Panthera zdanskyi* is estimated to have had a body mass of 76.8 kg.

A recent paper [1] analysed the relationship between narial aperture size and body mass in felids, and based on their formula we computed an estimated body mass for the paratype specimen of *Panthera zdanskyi*, since the narial aperture is nearly undistorted in this specimen, but parts of the anterior-most nasals are weathered away, making inference of narial aperture size tentative. Approximate nasial aperture area is ~1500 mm^2^, implying a body mass of around 134 kg. Although this specimen appears to have been slightly larger than the holotype, this value appears excessive, and the method is sensitive to fossil taxa with larger narial apertures to skull sizes than extant felids, e.g., many sabertoothed felids and the Eurasian cave lion (*Panthera spelaea*) which was found to have weighed in excess of 400 kg by [1], a value that appears excessive compared to the overall skeletal size of these animals relative to extant lions, which are known to weigh less than half this value [2]. *Panthera zdanskyi* would appear also to have had a rather large narial aperture, possibly inflating its body mass estimate based on this kind of data.

1. Torregosa V, Petrucci M, Pérez-Claros JA, Palmquist P (2010) Nasal aperture area and body mass in felids: ecophysiological implications and paleobiological inferences. Geobios 43: 653-661.

2. Sunquist M, Sunquist F (2003) Wild cats of the world. Chicago: Univ. Chicago Press.
