# Supplementary material for: Oldest Known Pantherine Skull and Evolution of the Tiger
Source: PLoS One. 2011 Oct 10;6(10):e25483. doi: 10.1371/journal.pone.0025483 (PMC3189913; doi:10.1371/journal.pone.0025483)
Supplement: Appendix S2 — Description of characters and data matrix used in phylogenetic analysis. For detail interpretation of character selection and coding, please see [1]. (DOC) [file pone.0025483.s008.doc]

**Appendix 2**. Cladistic analysis.

Description of characters and data matrix used in phylogenetic analysis. For detailed interpretation of character selection and coding, as well as references used, please see [1].

1: Longitudinal ridge along auditory meatus absent (0) or present (1). This character is tentative in *Panthera palaeosinensis*.

2: Processus muscularis on malleus short, cone-shaped and with tapering apex (0), or distinctly enlarged and cylindrical.

3: Processus brevis on malleus not enlarged or anteriorly deflected (0), or large and anteriorly deflected (1).

4: Inferior head of incus for articulation with malleus smooth and not enlarged (0), or distinctly enlarged (1).

5: Stylomastoid foramen groove: distinct groove consisting of a bony ridge of the ectotympanic and squamosal merging posteriorly with the mastoid process (0); lacking anteroventral projection of the mastoid process, so that the ectotympanic part of the bony ridge is separated by a fissure from the squamosal-mastoid part (1); posterior body ridge from ectotympanic fused with squamosal, forming a large continuous surface with the mastoid process (2) [un-ordered].

6: Frontal sinus situated in the anterior part of the postorbital region (0), or posterior to the postorbital region (1).

7: Posterior part of the first caudal ethmoturbinal scroll situated distinctly posterior to (0) or anterior to (1) the postorbital process.

8: Deciduous P^4^ with seperate (0) or fused (1) parastyle and metastyle roots.

9: Deciduous P^3^ with distinct, rounded and chisel-like accessory cusp (0) or with a reduced, flattened cusp (1).

10: Nasal-frontal suture extending to the level of maxillary-frontal suture, sometimes slightly posterior to it, but often terminating well anterior to it (0), or extending distinctly posterior to maxillary-frontal suture (1). In most snow leopards and many ocelots and lions, the nasal-frontal suture terminates well anterior to the maxillary frontal suture, whereas the two are at level in the other taxa scored as plesiomorphic.

11: Frontoparietal (coronal) suture distinctly posterior to postorbital constriction (0); or in some specimens posterior to but frequently close to postorbital constriction (1).

12: Lambdoidal-coronal sutures converging along occipital crest (0), or separate (1). There is some intraspecific variation, and in some specimens the sutures can be scored as converging, although they do not physically touch, thus excluding the parietal from the occipital crest; it is incorrect to state that in lions the sutures are unanimously widely separated, whereas in tigers they always converge.

13: Dorsal part of frontoparietal (coronal) suture smooth, lacking a parietal process (0); or suture irregular with a distinct, often very large, medio-dorsally directed parietal process (1). In some ocelots and lions, a parietal process may be present, but it is distinctly smaller than in the puma. The snow leopard often has not one, but two large, mediodorsal parietal projections.

14: Presence (0), occasional presence (1) or absence (2) of anteriorly directed bulge overhanging the infraorbital foramen [un-ordered]. In the puma and ocelot, an anterior bulge from the jugal-maxillary overhangs the infraorbital foramen, and in approximately half of clouded leopards, this is also the case, although the bulge is smaller. In the other great cats, this bulge is absent.

15: Infraorbital foramen always single (0) or occasionally double (1). In the lion, the infraorbital foramen is frequently double in the Asiatic lion (*Panthera leo persica*), and occasionally even triple, whereas all other lions have a single infraorbital foramen, although a double foramen may very rarely be present in African lions. In some clouded leopards, e.g. ZMB81359 or SSTM02104, the foramen is also double, although in most specimens it is single.

16: Bridge above preorbital foramen. Absent (0); present (1).

17: Lacrimal process. Absent (0); small (1); large (2) [un-ordered].

18: Dorsal process of maxilla wide and round (0), or truncated and tapering (1). This character is not coupled with the abbreviated, tall rostrum characteristic of non-pantherine felines, because the snow leopard also has this feature.

19: Jugal-squamosal suture in zygomatic arch does (0), sometimes does (1) or does not (2) abut the postorbital process [un-ordered]. In the puma and ocelot, and also in the Ice age *Panthera spelaea* and *P. atrox*, the suture is adjacent to the postorbital process. In the clouded leopard, leopard, and tiger, the suture abuts the postorbital process in around half the specimens and the gap is small in the other half, whereas other great cats have a distinct, frequently wide (0.5–1 cm) dorsal projection from the jugal separating the suture from the postorbital process. *P. palaeosinensis* has a narrow gap, and, accordingly, was scored 1, although this is tentative.

20: Zygomatic arch slender (0) or wide (1). See Fig. 1a.

21: Jugal postorbital process large (0); reduced (1). In the clouded leopard, some specimens have a moderately well developed postorbital process, but others have almost none; this condition is never observed among other pantherine felids.

22: Paroccipital process large and ventrally directed (0); reduced and posteriorly directed (1).

23: External profile of muzzle in dorsal view: tapering or gently concave (0); or gently convex (1).

This character is unrelated to the massiveness of the upper canine roots, as the lion has large, thick canines but a convex profile, implying gentle tapering of the muzzle posteriorly towards the infraorbital foramina, and anteriorly towards the premaxilla, whereas the clouded leopard has much more slender canines, but a concave profile, implying widening of the muzzle towards the premaxilla, as in the jaguar or tiger.

24: Nasal width at narial aperture: narrow (0); moderately wide (1); wide (2) [un-ordered].

25: Nasal width at frontal-maxillary suture: narrow (0); wide (1).

26: Nasals relatively short (0), or long (1).

27: Palate length: 0, relatively short (0); moderately long (1); long (2) [un-ordered].

28: Pterygoid palate relatively narrow (0); relatively wide (1).

29: Angle of long axis of bulla to long axis of skull: shallow (25–25^o^ [0]); 1: obtuse (>~40 ^o^ [1]).

30: Entotympanic process narrow and raised (0); or wide and flattened (1).

31: Projection of foramen rotundum: anteriorly (0); laterally (1).

32: Fossa for posterior lacerat foramen and condylar foramen oval or slit-like (0); or distinctly wider and triangular (1). In the Pantherinae, the condylar foramen is more posteromedially offset from the posterior lacerat foramen, creating a triangular fossa.

33: Stylomastoid foramen small and slit-like (0); or enlarged (1). In pumas and clouded leopards, the stylomastoid foramen is sometimes small, but it is usually distinctly enlarged over the condition in the ocelot. In *Panthera*, the foramen is usually distinctly enlarged, and more ventrally exposed than in small felids.

34: P^2^ always or nearly always present in at least one side of skull and unreduced in size (0); P^2^ reduced in size and frequently absent in both sides of skull (1). In most felids, P^2^ is present in at least one side of the skull, and most often in both. In the clouded leopard, P^2^ is reduced in size, frequently absent in one side of the skull, and in over one-third of specimens it is absent altogether. The alveoli for the permanent teeth are either empty or fused partly or completely. In *Panthera*, P^2^ is always present in the jaguar and snow leopard, and in leopards and lions, only around 1% of specimens lack P^2^ in both sides of the skull, whereas this is the case in around 5% of tigers.

35: P^3^ parastyle relative to P3 crown length: short (0); long (1).

36: P^4^ ectoparastyle weakly developed (0) or strongly developed (1). It is sometimes said that the tiger is characteristic among extant *Panthera* in having a well-developed ectoparastyle, but this is present in other species as well.

37: C^1^ relative to skull length: not elongate (0); elongate (1).

38: Proportions of C^1^ crown cross-section: oval (0); almost round (1); blade-like (2) [un-ordered].

39: Ventral profile of mandibular ramus: Concave (0); convex or straight (1); usually convex, but concave in some specimens (2) [unordered]. Some specimens of *Panthera atrox* and the jaguar deviate from the pattern observed among other *Panthera*, in that most specimens have a perfectly straight or gently concave ventral ramus profile, as in tigers, but several specimens have a markedly concave ramus profile, as in lions or leopards.

40: Angle of mandibular symphysis relative to long axis of horizontal ramus: symphysis distinctly (>~130 ^o^) anteriorly inclined (0); symphysis weakly anteriorly inclined (~110–120 ^o^ [1]). The clouded leopard has a more so-called verticalised mandibular symphysis than any other extant felid, approaching the condition among primitive sabretoothed felids.

41: Transition from horizontal mandibular ramus to mandibular symphysis: smoothly round (0); abrupt (1). In the jaguar, most specimens have an abrupt transition, resembling the tiger, although a

few specimens have a more smoothly rounded transition; it is, however, not as rounded as in, for instance, lions or pumas.

42: Mandibular symphysis without (0) or with (1) strong anterodorsal curvature along anterior part of dentary.

43: Mandible not taller (0) or taller (1) at the front (anterior to P_3_) than in middle (at M_1_ ⁄P_4_).

44: Length of M_1_ crown relative to mandible: not enlarged (0); enlarged (1). See Fig. 1i.

45: Length of C_1_ relative to mandibular length: not elongate (0); elongate (1).

46: Contracted pupil narrow and slit-like (0) or round (1); *Neofelis* has a less round pupil than *Panthera*, and it is often described as oval.

47: Medial face of claw sheath less developed than lateral face (0), or both faces equally developed (1).

48: Fully ossified hyoid, including epihyal portion (0), or partly fused hyoid with cartilagious epihyal portion (1). See Discussion in character 55.

49: Whorl of hair present between and behind ears (0); behind ears and extending down the neck (1); or between the shoulders (2) [unordered].

50: Mane absent (0) or occasionally (1) or usually (2) present [unordered]. Among extant felids, a prominent mane is a present in the male lion exclusively, although the mane is heavily influenced by both internal and external factors, such as climate; in hot climate zones, male lions can have but a short, thin mane, or none at all. Tigers have distinctly elongate cheek tufts, but occasionally tiger males will develop a true mane extending along the entire neck region towards the shoulders. In *Panthera spelaea*, even naturalistic cave art with clearly discernible male characteristics, such as a scrotum, does not depict this species with a mane.

51: Dorsal face of snout with fur not extending (0) or extending (1) all the way to the rhinarium.

52: Lateral sides of facial region and upper neck often supplied with lateral, longitudinal stripes (0), or either un-patterned or spotted (1). In the tiger, the autapomorphic stripe pattern is different from that of small felids, in that the stripes are primarily dorsoventral. A uniform, un-patterned coat pattern may be inferred in *Panthera spelaea* from naturalistic cave paintings.

53: Vocal repertory: prusten absent (0) or present (1).

54: Vocal repertory: puff vocalization absent (0) or present (1).

55: Vocal repertory: true roaring sequence absent (0) or present (1). Traditionally, the great cats (*Panthera*) have been termed roaring cats, owing to the presence of a cartilaginous epihyal portion of the hyoid, which is purported to permit true roaring. However, recent research has cast doubt on this, because only three of the five species of extant *Panthera* have an actual roaring sequence, and several species of small felids appear to have a roaring sequence, although not as powerful and much higher pitched, owing to their small body size. There is also doubt as to the correlation of a fully ossified epihyal and purring, since several authors maintain that at least some pantherine cats can purr.

56: Vocal repertoire: grunt absent (0) or present (1).

57: Feeding position slightly crouched (0), or lying down (1).

58: Position of tail when sitting down: wrapped around body (0); or extended posteriorly from body (1).

Data matrix

1111111111222222222233333333334444444444555555555

1234567890123456789012345678901234567890123456789012345678

*Leopardus pardalis* 0001001000000001000000000000000000000000000000000000000000

*Puma concolor* 0000000000001001100100021011001010100100100001000000000000

*Neofelis nebulosa* 1000200100000111111111011010110111001211111010100000100001

*Panthera leo*  1100210110110210112100121021111110110000000001112211011111

*Panthera onca* 1110210111110200212100021011110110110020100001112011101111

*Panthera pardus* 1100210110110100111100121021111110100000000001112011011111

*Panthera tigris*  1000210111100200211100021121110110110010100011111111100011

*Panthera uncia* 1011101110101200212100121011111110000101001111110011100001

*Panthera atrox* 1???2101?011020021010012102111111010002010000?????????????

*Panthera palaeosinensis* 1???2????010020021110000102111011011001110100?????????????

*Panthera spelaea* 1???21011011020011010012102111111011000010000????0?1??????

*Panthera zdanskyi* 1????1???110020011210002112?11011011001010011?????????????
